# Gonadotropin-releasing hormone-like receptor 2 inversely regulates somatic proteostasis and reproduction in *Caenorhabditis elegans*

**DOI:** 10.3389/fcell.2022.951199

**Published:** 2022-08-29

**Authors:** Mor Kishner, Libat Habaz, Lana Meshnik, Tomer Dvir Meidan, Alexandra Polonsky, Anat Ben-Zvi

**Affiliations:** Department of Life Sciences, Ben-Gurion University of the Negev, Beer Sheva, Israel

**Keywords:** aging, gonadal longevity signaling, gonadotropin-releasing hormone (GnRH/GnRH receptor), proteostasis, stress response, reproduction, *C. elegans*

## Abstract

The quality control machinery regulates the cellular proteome to ensure proper protein homeostasis (proteostasis). In *Caenorhabditis elegans*, quality control networks are downregulated cell-nonautonomously by the gonadal longevity pathway or metabolic signaling at the onset of reproduction. However, how signals are mediated between the gonad and the somatic tissues is not known. Gonadotropin-releasing hormone (GnRH)-like signaling functions in the interplay between development and reproduction and have conserved roles in regulating reproduction, metabolism, and stress. We, therefore, asked whether GnRH-like signaling is involved in proteostasis collapse at the onset of reproduction. Here, we examine whether *C. elegans* orthologues of GnRH receptors modulate heat shock survival. We find that *gnrr-2* is required for proteostasis remodeling in different somatic tissues during the transition to adulthood. We show that *gnrr-*2 likely functions in neurons downstream of the gonad in the gonadal-longevity pathway and modulate the somatic regulation of transcription factors HSF-1, DAF-16, and PQM-1. In parallel, *gnrr-2* modulates egg-laying rates, vitellogenin production, and thus reproductive capacity. Taken together, our data suggest that *gnrr-2* plays a GnRH-associated role, mediating the cross-talk between the reproduction system and the soma in the decision to commit to reproduction.

## Introduction

The age-dependent dysregulation of quality control machinery and, specifically, protein homeostasis (proteostasis), is associated with limited ability to mount stress responses, reduced folding capacity, accumulation of protein damage, and increased prevalence of protein misfolding diseases (Brehme et al., 2014; Vilchez et al., 2014; Huang et al., 2019; Morimoto, 2020; Taylor and Hetz, 2020; Aman et al., 2021; Meller and Shalgi, 2021; Shemesh et al., 2021). Transcriptional stress response programs, such as the heat shock response (HSR) and the unfolded protein responses in the ER and mitochondria (UPR^ER^ and UPR^mt^, respectively), are remodeled at the onset of *Caenorhabditis elegans* reproduction and in human senescent cells (Shai et al., 2014; Li et al., 2017; Taylor and Hetz, 2020; Meller and Shalgi, 2021; Sala and Morimoto, 2022). While many cytoprotective genes are upregulated under stress conditions in young adults or primary human fibroblasts, their activation is impaired in reproductive adults and senescent cells, leading to a sharp decline in stress survival (Ben-Zvi et al., 2009; Lapierre et al., 2011; Vilchez et al., 2012; Shemesh et al., 2013; Taylor and Dillin, 2013; Labbadia and Morimoto, 2015; Steinbaugh et al., 2015; Sabath et al., 2020). In *C. elegans,* HSR dysregulation is associated with a repressive chromatin state and reduced transcription of heat shock (HS) genes (Shemesh et al., 2013; Labbadia and Morimoto, 2015). Similar modifications at the chromatin state, including induction of repressive marks and reduction of activating marks, were noted in other aging organisms and senescent human cells (Meller and Shalgi, 2021; Sala and Morimoto, 2022). These findings support a broad role for stress response pathways remodeling in the age-dependent regulation of proteostasis.

Work in *C. elegans* demonstrates that reproductive competence and environmental conditions could modulate the timing of the proteostasis decline (Ben-Zvi et al., 2009; Lapierre et al., 2011; Vilchez et al., 2012; Shemesh et al., 2013; Taylor and Dillin, 2013; Labbadia and Morimoto, 2015; Shemesh et al., 2017a; Shemesh et al., 2017b; Labbadia et al., 2017; Matai et al., 2019; Shpigel et al., 2019; Sala et al., 2020). For example, disrupting germline proliferation results in the remodeling of many stress transcriptional programs, leading to maintenance of robust proteostasis while inversely modulating reproduction (Berman and Kenyon, 2006; Gerisch et al., 2007; Goudeau et al., 2011; Lapierre et al., 2011; Vilchez et al., 2012; Shemesh et al., 2013; Taylor and Dillin, 2013; Tepper et al., 2013; Labbadia and Morimoto, 2015; Steinbaugh et al., 2015; Nakamura et al., 2016; Cohen-Berkman et al., 2020). In contrast, food limitation and dietary restriction (DR) could reverse the collapse and restore the activation of the HSR even late in adulthood (Thondamal et al., 2014; Matai et al., 2019; Shpigel et al., 2019). Proteostasis collapse is therefore regulated non-autonomously in response to life history events and environmental changes. But how are these signals transmitted and integrated between tissues to mediate proteostasis collapse in the soma?

The gonadotropin-releasing hormone (GnRH) superfamily acts at the interplay between development and reproduction. This superfamily integrates internal and environmental stimuli to regulate sexual maturation and reproductive functions in vertebrates and invertebrates (Zandawala et al., 2018; Sakai et al., 2020). This G protein-coupled receptor (GPCR) superfamily is subdivided into two main subfamilies based on neuropeptides sequence conservation. GnRH receptors subfamily, including GnRH, Adipokinetic hormone (AKH), and AKH-CRZ–related peptide (ACP) binding receptors, and Corazonin (CRZ) receptors subfamily (Zandawala et al., 2018). The genome of *C. elegans* encodes eight members of the GnRH-like GPCRs superfamily (*gnrr-1* to *gnrr-8*; GNRRs) (Van der Auwera et al., 2020), the ligands of four were identified. Of the four, only *gnrr-1*, an AKH-like receptor, and its neuropeptide, *nlp-47*, regulate fecundity (Lindemans et al., 2009). Here, we asked whether *C. elegans* GnRH-like receptors could remodel proteostasis at the onset of reproduction. We identify two family members, *gnrr-2* and *gnrr-6*, as putative regulators of proteostasis collapse. We focus on *gnrr-2* and demonstrate that disrupting *gnrr-2* function or expression leads to the maintenance of robust proteostasis in adulthood. We show that *gnrr-2* acts in the gonadal longevity pathway downstream of the gonad and inversely modulates reproduction, suggesting that *gnrr-2* functions as a GnRH-like receptor.

## Materials and methods

### Nematode strains and growth conditions

A list of strains used in this work is provided in Supplementary Table S1. Mutant strains were outcrossed into our N2 strain (*n* ≥ 3). Standard genetic crossing techniques were used to construct mutant strains and mutation were verify using single worm PCR (Phire Animal Tissue Direct PCR Kit, Thermo Scientific) as previously described (Meshnik et al., 2022). Nematodes were cultured using standard techniques. Animals were grown on NGM plates seeded with the *Escherichia coli* OP50-1 strain at 15°C. For RNA interference (RNAi), eggs were placed on *E. coli* strain HT115 (DE3) transformed with specified RNAi or empty vector (pL4440) control (obtained from the Ahringer or Vidal RNAi libraries). RNAi efficiency was determined using qPCR to determine the mRNA levels, as in (Dror et al., 2020). For diet supplementation of fatty acids, plates were supplemented with the detergent Tergitol (NP40; Sigma) used as control, or with AA (50 µM TCI Chemical dissolved in NP40), as in (Shemesh et al., 2017a). Unless otherwise stated, eggs, laid at 15°C, were transferred to fresh plates and grown at 25°C for the duration of an experiment. The first day of adulthood was set at 50 h after temperature shift, before the onset of egg-laying. To avoid progeny contamination, animals were moved to fresh plates during the reproductive period.

### Statistical analyses

To examine whether any *gnrr* family member improved HS survival rates compared with wild type (WT) animals, we used a one-way analysis of variance (ANOVA) followed by a Dunnett’s *post-hoc* test. To test the null hypothesis that *gnrr-2* modulated proteostasis capacity after the onset of reproduction, we used one-way ANOVA followed by a Tukey’s *post-hoc* test. We used the same test to examine the impact of *gnrr-2* on reproduction. To compare proteostasis or reproduction capacity between two strains or two RNAi treatments, we used two-tailed Wilcoxon Mann-Whitney rank-sum test. To compare the expression levels of genes and assess their statistical significance, we used the Wilcoxon Mann-Whitney rank-sum test; Bonferroni correction was applied to adjust *p* values when gene expression was also compared with *glp-1* as a positive control. Mean life spans were calculated using Kaplan-Meier survival curves and were compared using Mantel-Cox log-rank test. Data are means ±1 standard error of the mean (1 SE). Unless otherwise indicated, (∗) denotes *p* ≤ 0.05, and (∗∗) denotes *p* ≤ 0.01. (*N*) denotes the numbers of biological repeats, and (*n*) denotes the number individuals per experimental condition.

### *gnrr-2* deletion mutants

Genomic DNA from WT or *gnrr-2*(*ok3618*) animals was amplified with a single worm PCR Phire Animal Tissue Direct PCR Kit (Thermo Scientific) and sequenced (IDT) using primers flanking the *ok3618* deletion (Supplementary Table S2). A 417 bp deletion was identified (positions 2515-2931 of the C15H11.2a transcript), spanning *gnrr-2* exon 6, intron 6, exon 7, and its’ 3′ UTR (Supplementary Figure S1). The deletion also partially disrupts the 3′ UTR of the nuclear export factor, *nxf-1* (*C15H11.3*), encoded on the opposite strand (Supplementary Figure S1). The *gnrr-2*(*tm4867*) deletion was previously characterized (Consortium, 2012). It is a 477 bp deletion spanning exons 4–6 not affecting *nxf-1* (Supplementary Figure S1). Because *nxf-1* is an essential gene involved in mRNA export from the nucleus, and mutations in *nxf-1* result in embryonic arrest and lethality (Zheleva et al., 2019), we estimate little to no effect of the *ok3618* deletion on its’ function*.* This conclusion is further supported by the consistent results observed for the two deletion strains, and *gnrr-2* RNAi, strongly reducing the possibility that *nxf-1* contributes to the phenotypes reported.

### Heat shock assays

HS survival rates were determined as previously described (Karady et al., 2013). Briefly, age-synchronized animals were subjected to 37°C for 6 h, unless otherwise indicated, and survival was scored by monitoring SYTOX orange dye uptake (*N* ≥ 3, *n* > 100). Fluorescent animals were scored as dead. For HS activation assays, plates with age-synchronized animals (*N* ≥ 5) were placed in a 37°C bath for 90 min. Animals were frozen immediately following the HS. GFP_HS_-expressing animals (*N* ≥ 3, *n* ≥ 60) were fixed 18–24 h following the HS and imaged using a Leica DM5500 confocal microscope through a 40x 1.0 numerical aperture objective with 488 nm laser line for excitation as previously described (Shpigel et al., 2019). Animals expressing GFP in the gut were scored as HS-induced. Alternately, images were analyzed using the ImageJ software (NIH), and GFP levels were determined. For HS recovery assays, age-synchronized animals were subjected to 37°C for 4 h, and recovery was scored by monitoring motility 4 h after the HS (*N* ≥ 5, *n* > 125). This assay was used to score TU3401 animals that express mCherry and thus cannot be scored with SYTOX orange.

### Determination of RNA levels

RNA extraction, cDNA synthesis, and quantitative real-time PCR were performed as previously described (Shemesh et al., 2017a). Samples (*N* ≥ 5) were normalized to *act-1* using the 2-ΔΔCT method. Samples were also normalized to 18S to verify that *act-1* is not modulated under these experimental conditions, and the results were consistent. A list of primers is provided in Supplementary Table S2.

### Foci quantification

Age-synchronized animals (*N* ≥ 3, *n* > 30) expressing *punc-54*::Q35::YFP, were imaged using a Leica M165 FC fluorescent stereoscope with a YFP filter. The number of bright foci, discrete structures that are brighter than the surrounding fluorescence, was counted.

### Motility assays

For thrashing rates, age-synchronized animals (*N* ≥ 3, *n* ≥ 40) were monitored, and thrashes (changes in bending direction at mid-body) were counted, as in (Dror et al., 2020). Values are presented as bends per minute. For Stiff-body paralysis, age-synchronized *unc-52*(*ts*) mutant animals (*N* ≥ 12, *n* > 100) grown at 25°C until day one of adulthood were shifted to 15°C. Motility was scored by monitoring animal movement 10 min after transfer to a new plate on day 4 of adulthood, as in (Shemesh et al., 2013). Animals that did not move were scored as paralyzed.

### DAF-16 and PQM-1 nuclear localization assay

Age-synchronized DAF-16::GFP or PQM-1::GFP animals (*N* ≥ 3 and *n* = 10) were grown at 25°C until day two of adulthood. Animals were fixed, mounted, and imaged using a Leica DM5500 confocal microscope through a 40x 1.0 numerical aperture objective with 488 nm laser line for excitation as previously described (Shemesh et al., 2017a). Animals showing nuclear-localized GFP in the majority of their intestinal cells were scored as positive. Animals were scored blind.

### *gnrr-2* localization assay

The promoter region of *gnrr-2* (1,040 bp upstream of the protein-coding region) was amplified from the genomic DNA of WT animals, cloned into pNU435 plasmid using the Gibson Assembly method (Macrogen) and verified by sequencing. All primers are listed in Supplementary Table S2. This plasmid was co-injected with a marker plasmid expressing *pmyo-3*::*mCherry* into WT animals and maintained as an extra-chromosomal array. Age-synchronized animals expressing *gnrr-2p*::*GFP* and myo-3p::mCherry were grown at 25°C. Animals were fixed at the indicated stages using paraformaldehyde (4%) and mounted on microscope slides. *gnrr-2p*::*GFP* and *myo-3p*::*mCherry* were imaged using a Leica DM5500 confocal microscope through a 40x 1.0 numerical aperture objective with 488 nm and 532 laser lines, respectively, for excitation.

### Progeny quantification

Age-synchronized animals (*N* ≥ 3, *n* ≥ 18) were allowed to lay eggs on seeded plates (one animal per plate). Animals were moved to freshly seeded plates every day until the end of the reproductive period, and the progeny number of each animal was scored 24–48 h later, as previously described (Shemesh et al., 2017b).

### Eggs laying rate

Age-synchronized day two adults (10 animals per plate; *N* ≥ 7, *n* > 100) were allowed to lay eggs on seeded plates. Animals were moved every hour, and the progeny number was scored.

### Yolk protein YP170 quantification

Similar numbers of age-synchronized day two adult animals were collected and lysed in SDS sample buffer (92°C for 10 min). Sample (equal volumes) were loaded on 8% SDS-PAGE and separated using gel electrophoresis. Gels were stained using Coomassie brilliant blue and imaged using a ChemiDoc™ MP Imaging System (Bio-Rad Laboratories). The YP170 band was identified by comparing to previous publications (DePina et al., 2011; Plagens et al., 2021), and compared with *glp-1* mutant animals that accumulate YP (Steinbaugh et al., 2015). Images were analyzed using the ImageJ software (NIH).

### Oil-Red-O staining

Animals were fixed and stained as previously described (O’Rourke et al., 2009). Animals were then imaged using a Leica DMIL microscope with a 10x 1.0 objective. Images were analyzed using the ImageJ software (NIH).

### Lifespan analysis

∼130 animals were monitored for each strain starting from day one of adulthood (10–15 animals per plate), as previously described (Shemesh et al., 2017a).

### Embryo hatching

Embryos (*N* ≥ 4, *n* > 100) were set on a freshly seeded plate, and hatching was examined after 24 or 48 h using a Leica M165 FC stereoscope.

### Developmental timing

Embryos were grown at 20°C, animals’ developmental stage was examined daily, and the number of reproductive adults was recorded, as previously described (Dror et al., 2020).

## Results

### Examining the role of GNRR gene family in proteostasis remodeling

**FIGURE 1:**
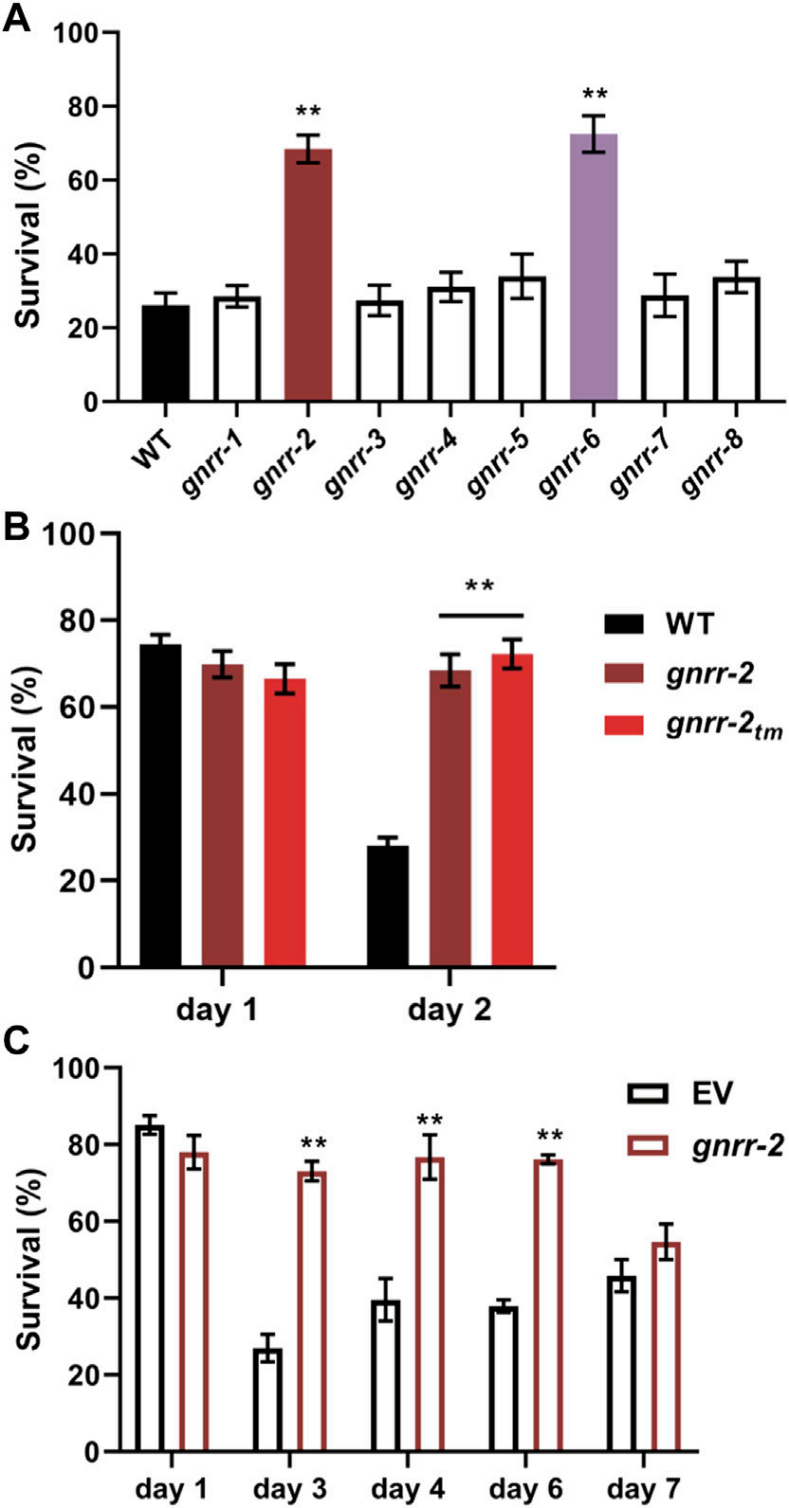
Gonadotropin-related hormone receptors *gnrr-2* and *gnrr-6* modulate HS survival in adulthood. **(A)** HS Survival rates of age-synchronized WT or *gnrr-1* to *gnrr-8* mutant animals. Animals were subjected to HS (6 h at 37°C) on day two of adulthood, and survival was assayed (*N* ≥ 5). Data are means ±1 standard error of the mean (1 SE). Data were analyzed using one-way ANOVA followed by a Dunnett’s *post-hoc* test. (**) denotes *p* ≤ 0.001 compared with WT animals. **(B)** HS Survival rates of age-synchronized WT or mutant animals, *gnrr-2* and *gnrr-2*_*tm*_. Animals were subjected to HS (6 h at 37°C) on day one or two of adulthood, and survival was assayed (*N* ≥ 5). Data are means ±1 standard error of the mean (1 SE). Data were analyzed using one-way ANOVA followed by a Tukey’s *post-hoc* test. (**) denotes *p* ≤ 0.001 compared with same-age WT animals. **(C)** HS Survival rates of age-synchronized WT, fed on empty vector (EV) or *gnrr-2* RNAi-expressing bacteria. Animals were subjected to HS (6 h at 37°C) on days 1–7 of adulthood, as indicated, and survival was assayed (*N* ≥ 5). Data are means ±1 standard error of the mean (1 SE). Data were analyzed using one-way ANOVA followed by a Tukey’s *post-hoc* test. (**) denotes *p* ≤ 0.001 compared with same-age animals fed on EV RNAi.

To ask whether GNRRs family members play a role in age-dependent proteostasis remodeling, we examine whether mutant animals in each *gnrr* gene (Supplementary Table S1) could rescue the sharp decline in HS survival rates at the onset of reproduction. Survival rates of *gnrr-1*, *gnrr-3*, *gnrr-4*, *gnrr-5*, *gnrr-7*, and *gnrr-8* mutant animals (6 h at 37°C, day two adults) were similar to WT. In contrast, survival rates of *gnrr-2*(*ok3618*) (hereon named *gnrr-2*; Supplementary Figure S1) and *gnrr-6*(*ok3362*) mutant animals were significantly improved (66% ± 3% and 72% + 5%, respectively, ANOVA followed by a Dunnett’s post-hoc test, *p* ≤ 0.001; Figure 1A). Because GNRR-6 and GNRR-3 are activated by opposing RPamide neuropeptides NLP-22 and NLP-2 and promote sleep and wakefulness, respectively (Van der Auwera et al., 2020), we focused on GNRR-2 and examined the role of this GPCR in proteostasis remodeling.

To validate *gnrr-2* role in HS survival, we tested the thermotolerance of a second *gnrr-2* allele (see *Materials and methods*; Supplementary Figure S1) and used RNA interference (RNAi) to knock down *gnrr-2* expression. We observed increased survival rates for *gnrr-2*(*tm4867*) (hereon named *gnrr-2*_*tm*_) mutant animals following HS (72% ± 3%, 6 h at 37°C, day two adults, ANOVA followed by a Tukey’s post-hoc test, *p* ≤ 0.001; Figure 1B). Likewise, HS survival rates of WT animals treated with *gnrr-2*(*RNAi*) were strongly improved compared to animals treated with empty vector (EV) control (6 h at 37°C, day three adults, 73% ± 3% and 28% ± 4%, respectively, ANOVA followed by a Tukey’s post-hoc test, *p* ≤ 0.001; Figure 1C). Thus, *gnrr-2* dysfunction or downregulation improved thermotolerance in adulthood.

To ask whether *gnrr-2* modulates thermotolerance in general or specifically during adulthood, we compared the thermotolerance of WT and *gnrr-2* mutant animals before the collapse. HS survival rates of *gnrr-2* or *gnrr-2*_*tm*_ young adults (6 h at 37°C, day one adults) were not significantly different from WT animals (70% ± 3%, 67% ± 3%, and 74% ± 3%, respectively, ANOVA followed by a Tukey’s post-hoc test; Figure 1B). We found similar HS survival rates for WT young adults treated with *gnrr-2(RNAi)* or EV (78% ± 5% and 85% + 3%, respectively, ANOVA followed by a Tukey’s post-hoc test; Figure 1C). Finally, survival rates of WT animals treated with *gnrr-2*(*RNAi*) remained high during adulthood (73% ± 2% for day six adults; Figure 1C), similar to the activation of gonadal longevity signaling (Shemesh et al., 2013). These data suggest that *gnrr-2* is required for remodeling thermotolerance at the transition to adulthood.

### *gnrr-2* modulates proteostasis during adulthood

**FIGURE 2:**
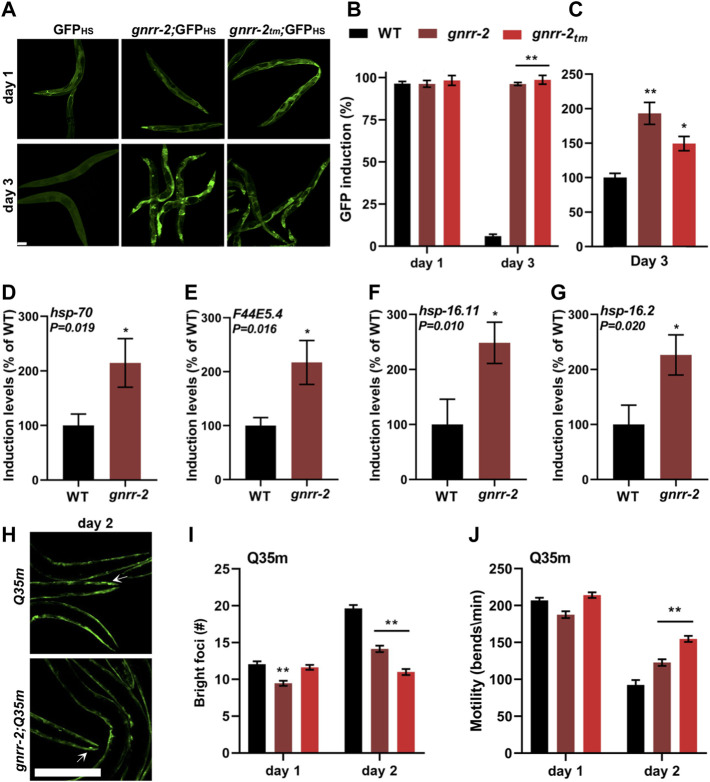
*gnrr-2* modulates HS response activation in adulthood. **(A–C)** HS-regulated GFP_HS_ expression. Age-synchronized WT, *gnrr-2*, and *gnrr-2*_*tm*_ animals expressing GFP_HS_ were imaged following a short HS 90 min at 37°C **(A)**. The percent of animals showing GFP was scored *N* ≥ 3 **(B)**, and GFP fluorescence levels (day three adults; *n* > 10) were determined **(C)**. Data are means ±1 standard error of the mean (1 SE). Data were analyzed using one-way ANOVA followed by a Tukey’s *post-hoc* test. (**) denotes *p* < 0.001 compared with same-age WT animals. The scale bar is 100 µm. **(D–G)** Expression levels of HS genes. mRNA levels of *hsp-70* **(D)**, *F44E5.4* **(E)**, *hsp-16.11* **(F)**, and *hsp-16.2* **(G)** from age-synchronized WT or *gnrr-*2 day two adults subjected to HS (90 min at 37°C; *N* ≥ 6). Data are means ±1 standard error of the mean (1 SE). Data were analyzed using the Wilcoxon Mann-Whitney rank sum test (*p* ≤ 0.020). **(H–J)** PolyQ foci number and motility in age-synchronized WT, *gnrr-2*, or *gnrr-2*_*tm*_ animals expressing *Q35m*. Age-synchronized *Q35m* expressing animals were imaged on day two of adulthood **(H)**. The scale bar is 250 µm, and arrows indicate foci. The number of visible foci *n* ≥ 33 **(I)** or thrashing rates *n* ≥ 35 **(J)** were scored on day one or two of adulthood. Data are means ±1 standard error of the mean (1 SE). Data were analyzed using one-way ANOVA followed by a Tukey’s *post-hoc* test. (**) denotes *p* < 0.001 compared with same-age Q35m animals.

WT animals cannot strongly induce the expression of HS genes after the onset of reproduction (Shemesh et al., 2013; Labbadia and Morimoto, 2015). To determine whether the improved thermotolerance of *gnrr-2* mutant animals is associated with HSR activation, we compared the ability of WT and *gnrr-2* mutant animals to mount an effective stress response. For that, we first monitored the expression pattern of a transcriptional reporter in which an *hsp-16.2* promoter regulates the expression of green fluorescent protein (GFP_HS_). We subjected WT and *gnrr-2* mutant animals carrying GFP_HS_ to HS (90 min at 37°C) on day one or three of adulthood and determined the percent of animals expressing GFP_HS_. While WT, *gnrr-2* and *gnrr-2*_*tm*_ day one adults showed induced GFP_HS_, only *gnrr-2* and *gnrr-2*_*tm*_ showed strong GFP_HS_ expression on day three of adulthood (Figures 2A,B). We detected expression in various somatic tissues with the most robust GFP_HS_ induction in the intestine (1.5-2-fold; Figures 2A,C). We next compared the expression levels of four HS genes between WT and *gnrr-2* day two adults following HS (90 min at 37°C). The mRNA levels of *hsp-70*, *F44E5.4*, *hsp-16.11*, and *hsp-16.2* were 2-fold higher in *gnrr-2* mutant animals than in WT animals (Wilcoxon Mann-Whitney rank sum test, *p* ≤ 0.02; Figures 2D–G). Higher levels were not due to improved HSR activation. HS induction of these genes was reduced in WT between day two and day one adults (Wilcoxon Mann-Whitney rank sum test, *p* ≤ 0.037; Supplementary Figures S2A–D), as previously demonstrated (Shemesh et al., 2013; Labbadia and Morimoto, 2015). In contrast, their expression was similarly induced in *gnrr-2* day one and two adults (*F44E5.4* expression improved; Supplementary Figures S2E–H). Thus, *gnrr-2* mutant animals maintain the ability to mount an effective HSR after the onset of reproduction rather than modulate HS activation efficacy.

The cell ability to maintain proteostasis in the face of chronic expression of misfolded proteins also declines with age (Ben-Zvi et al., 2009; Shemesh et al., 2013; Huang et al., 2019). To determine whether *gnrr-2* can modulate the accumulation and toxicity of misfolded proteins, we employed two polyQ protein aggregation models. Animals expressing 35 or 40 glutamine-repeats fused to a fluorescent protein in body-wall muscles (Q35m) or neurons (Q40n), respectively. Animals were crossed with *gnrr-2* or *gnrr-2*_*tm*_ mutant animals, and foci accumulation or toxicity were monitored. There were fewer bright foci in *gnrr-2*;Q35m and *gnrr-2*_*tm*_;Q35m compared to same-age Q35m animals (14 ± 1 and 11 ± 1 compared to 21 ± 1, ANOVA followed by a Tukey’s post-hoc test, *p* ≤ 0.001; Figures 2H,I). In agreement, the motility of *gnrr-2*;Q35m, *gnrr-2*_*tm*_;Q35m, and *gnrr-2*;Q40n animals, measured as thrashing rates, was more than 1.3-fold improved compared to Q35m and Q40n day two adults, respectively (ANOVA followed by a Tukey’s post-hoc test, *p* ≤ 0.001; Figure 2J; Supplementary Figure S2I). We also observed reduced foci number and improved motility rates when Q35m animals were treated with *gnrr-2*(*RNAi*) compared to EV control (ANOVA followed by a Tukey’s post-hoc test, *p* ≤ 0.001; Supplementary Figures S2J–L). Finally, we examined two well-characterized folding reporters*.* Temperature-sensitive missense mutations in *unc-45*(*e286ts*) and *unc-52*(*e669*, *su250ts*) destabilize myofilament folding and anchoring, respectively, leading to age-dependent motility defects. Motility of *gnrr-2*;*unc-45*(*ts*) animals, measured as thrashing rate, was more than 2.2-fold improved compared to *unc-45*(*ts*) day two adults. Likewise, motility of animals expressing *unc-52*(*ts*) was 1.8-fold improved in day four adults treated with *gnrr-2*(*RNAi*) compared to EV control (Wilcoxon Mann-Whitney rank sum test; Supplementary Figures S2M–N). Taken together, we find that disrupting *gnrr-2* function or expression led to improved proteostasis during aging.

### *gnrr-2* functions in the gonadal longevity pathway

**FIGURE 3:**
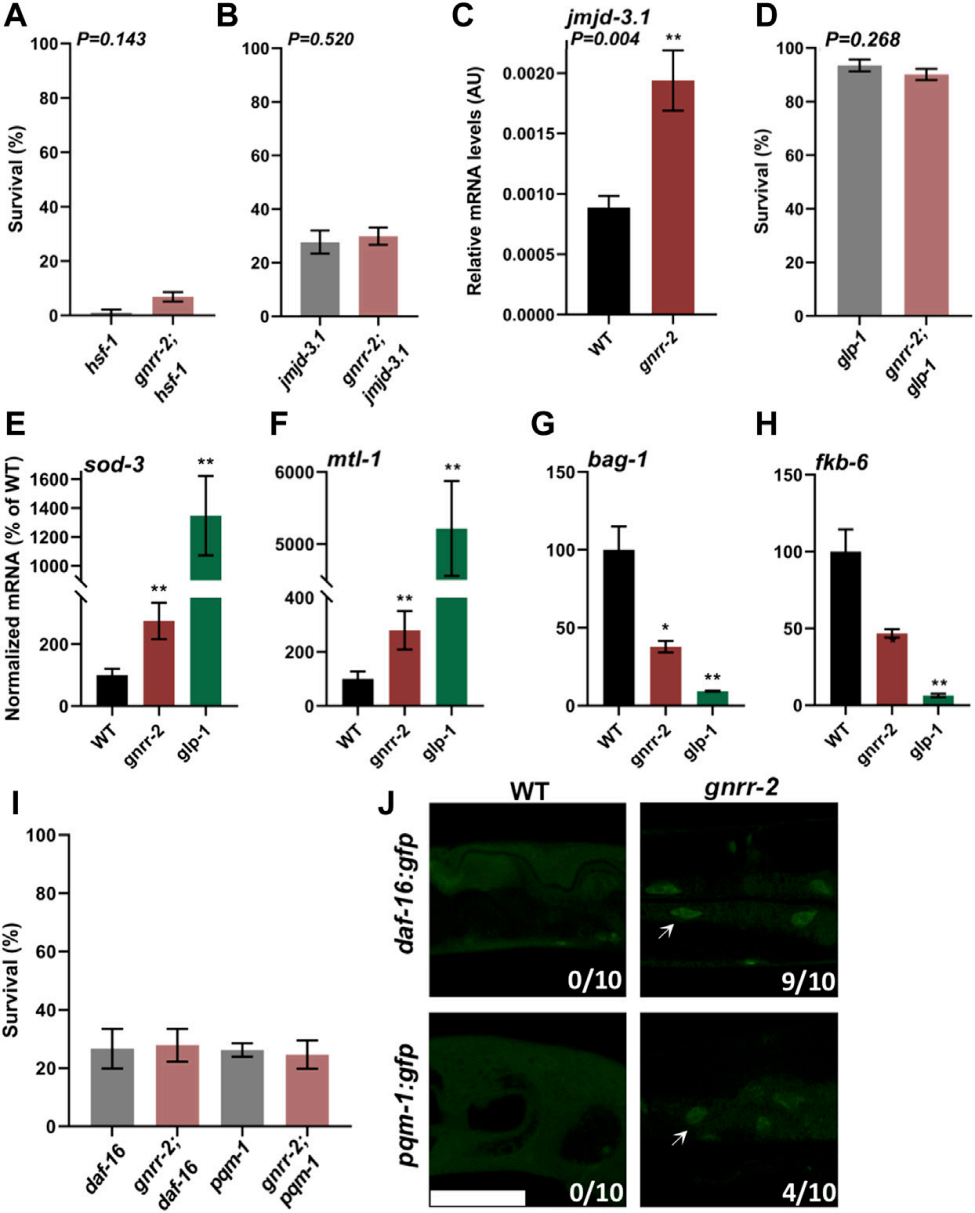
*gnrr-2* requires HSF-1, DAF-16, and PQM-1 to modulate somatic proteostasis. **(A,B)** HS Survival rates of age-synchronized *hsf-1(sy441)* and *gnrr-2;hsf-1* **(A)** or *jmjd-3.1(gk384)* and *gnrr-2;jmjd-3.1* **(B)** mutant animals. Animals grown at 15°C **(A)** or 25°C **(B)** were subjected to HS (6 h at 37°C) on day two of adulthood, and survival was assayed (*N* ≥ 6). Data are means ±1 standard error of the mean (1 SE). Data were analyzed using the Wilcoxon Mann-Whitney rank sum test (*p* = 0.143 and *p* = 0.520, respectively). **(C)** *jmjd-3.1* expression levels in age-synchronized WT or *gnrr-2* animals. mRNA was extracted from day two adult animals, and *jmjd-3.1* mRNA levels were quantified (*N* ≥ 5). Data are means ±1 standard error of the mean (1 SE). Data were analyzed using the Wilcoxon Mann-Whitney rank sum test (*p* = 0.004). **(D)** HS Survival rates of age-synchronized *glp-1*(*e2144*) or *gnrr-2*;*glp-1* mutant animals. Animals were subjected to HS (6 h at 37°C) on day two of adulthood, and survival was assayed (*N* ≥ 5). Data are means ±1 standard error of the mean (1 SE). Data were analyzed using the Wilcoxon Mann-Whitney rank sum test (*p* = 0.268). **(E–H)** Expression levels of DAF-16 or PQM-1 targets in age-synchronized WT, *gnrr-2*, or *glp-1* animals. mRNA was extracted from day two adult animals, and mRNA levels of *sod-3* **(E)**, *mtl-1* **(F)**, *bag-1* **(G)**, and *fkb-6* **(H)** were quantified (*N* ≥ 5). Data are means ±1 standard error of the mean (1 SE). Data were analyzed using the Wilcoxon Mann-Whitney rank-sum test with Bonferroni correction. (*) denotes *p* ≤ 0.01 (**) denotes *p* ≤ 0.003 compared with same-age WT animals. **(I)** HS Survival rates of age-synchronized *pqm-1*(*ok485*) or *daf-16*(*mu86*) mutant animals in a WT or *gnrr-2* background. Animals were subjected to HS (6 h at 37°C), and survival was assayed on day two of adulthood (*N* ≥ 4). Data are means ±1 standard error of the mean (1 SE). Data were analyzed using one-way ANOVA followed by a Tukey’s *post-hoc* test. **(J)** Representative images of age-synchronized animals expressing DAF-16::GFP or PQM-1::GFP in a WT or *gnrr-2* background. Day two adults were fixed, and the percentage of animals showing nuclear localization (arrows) was scored (*n* = 10 animals). The scale bar is 50 µm.

The decline in stress response activation after the onset of reproduction is linked to a repressed chromatin state at the promoters of HS genes. Specifically, HSF-1 transcriptional activation requires the H3K27 demethylase, JMJD-3.1, and its levels decline in WT animals at the transition to reproductive adulthood (Labbadia and Morimoto, 2015). To ask whether HSF-1 and JMJD-3.1 are required to maintain thermotolerance in *gnrr-2* mutant animals, we crossed *gnrr-2 with hsf-1*(*sy441*) or *jmjd-3.1*(*gk384*) mutant animals and examined their HS survival on day two of adulthood. As expected, we observed reduced survival rates for both *hsf-1* and *gnrr-2;hsf-1* mutant animals (4% ± 3% and 7% ± 2%, respectively, Wilcoxon Mann-Whitney rank sum test; Figure 3A). Likewise, HS survival rates of *gnrr-2*;*jmjd-3.1* double mutant animals were strongly reduced compared to *gnrr-2* (30% ± 3% compared to 66% ± 3%, respectively) and similar to *jmjd-3.1* single mutant (28% ± 4%, Wilcoxon Mann-Whitney rank sum test; Figure 3B). Activation of the gonadal longevity pathway restores *jmjd-3.1* levels (Labbadia and Morimoto, 2015; Shemesh et al., 2017a), while HS remodeling by dietary restriction does not (Shpigel et al., 2019). In agreement, the levels of *jmjd-3.1* mRNA on day two of adulthood in *gnrr-2* mutant animals were 2-fold higher than in WT animals (Wilcoxon Mann-Whitney rank sum test, *p* = 0.004; Figure 3C). These data suggest that *hsf-1* and *jmjd-3.1* are regulated by *gnrr-2* and support a role for *gnrr-2* in the gonadal longevity pathway.

Inhibition of germline stem cells (GSC) proliferation activates the gonadal longevity pathway and remodels proteostasis (Berman and Kenyon, 2006; Vilchez et al., 2012; Shemesh et al., 2013). To further examine whether *gnrr-2* acts *via* this pathway, we crossed *gnrr-2* mutant animals with *glp-1*(*e2141*), germline proliferation mutant animals, and monitored thermotolerance. The survival rate of the double mutants *gnrr-2*;*glp-1* was similar to that of *glp-1* mutant animals (6 h at 37°C; day two adults; 90% ± 2% and 93% ± 2%, respectively, Wilcoxon Mann-Whitney rank sum test; Figure 3D) and higher than *gnrr-2* single mutants. We observed similar behavior for *gnrr-2*(*RNAi*) treated animals even in prolonged HS (Supplementary Figure S3A). These data further support a role for *gnrr-2* in the gonadal longevity pathway. However, the lifespan of *gnrr-2* mutant animals was similar to WT (log-rank Mantel-Cox test, *p* = 0.066; Supplementary Figure S3B), as opposed to *glp-1* (Arantes-Oliveira et al., 2002). We, therefore, suggest that disrupting *gnrr-2* modulated some of the transcriptional pathways induced by germline loss.

GSC arrest triggers several transcriptional pathways associated with stress and metabolism, including DAF-16, DAF-12, SKN-1, PHA-4, HLH-30, and NHR-80 that are activated directly or as a result of downstream effectors (Berman and Kenyon, 2006; Gerisch et al., 2007; Goudeau et al., 2011; Lapierre et al., 2011; Tepper et al., 2013; Steinbaugh et al., 2015; Nakamura et al., 2016). To determine which transcription factors in the gonadal signaling pathway *gnrr-2* activates, we monitored the levels of target genes regulated by these downstream transcription factors after the onset of reproduction using qPCR. Of the eleven differentially regulated genes in germline proliferation mutant animals, only mRNA levels regulated by DAF-16 (*sod-1* and *mtl-1*) and PQM-1 (*bag-1* and *fkb-6*) were significantly modulated in *gnrr-2* day two adults (Wilcoxon Mann-Whitney rank-sum test with Bonferroni correction; Figures 3E–H; Supplementary Figures S3C–I)*.* The increase in *sod-1* and *mtl-1* expression and decrease in *bag-1* and *fkb-6* expression in *gnrr-2* compared to WT day two adults were not affected by HS (90 min at 37°C; Supplementary Figures S3J–M). Moreover, crossing *daf-16*(*mu86*) or *pqm-1*(*ok485*) mutants with *gnrr-2* mutant animals abolished their thermotolerance (6 h at 37°C; day two adults; 27% ± 5% and 25% ± 5%, respectively, ANOVA followed by a Tukey’s post-hoc test; Figure 3I). Monitoring the localization of DAF-16 or PQM-1 tagged with GFP showed that DAF-16 mainly localized to the nucleus in *gnrr-2* mutant animals, while PQM-1 only partially localized to the nucleus of *gnrr-2* mutant animals (Figure 3J). These data suggest that GNRR-2 regulates DAF-16 and PQM-1.

To ask whether *gnrr-2* regulates DAF-16 and PQM-1 specifically during adulthood, we compared the expression of *sod-1*, *mtl-1*, *bag-1*, and *fkb-6* in WT and *gnrr-2* mutants before the collapse. The expression levels of these four genes were not significantly different between *gnrr-2* and WT young adults (Wilcoxon Mann-Whitney rank sum test; Supplementary Figures S3N–Q). Of note, *gnrr-2* expression is upregulated in DAF-16-dependent manner, and *gnrr-2* promoter has a putative DAF-16 binding element (Tepper et al., 2013), suggesting that DAF-16 could itself modulate *gnrr-2* expression. Our data, therefore, suggest that GNRR-2 functions in the gonadal longevity pathway and the somatic regulation of DAF-16 and PQM-1.

### *gnrr-2* functions downstream of the gonad

**FIGURE 4:**
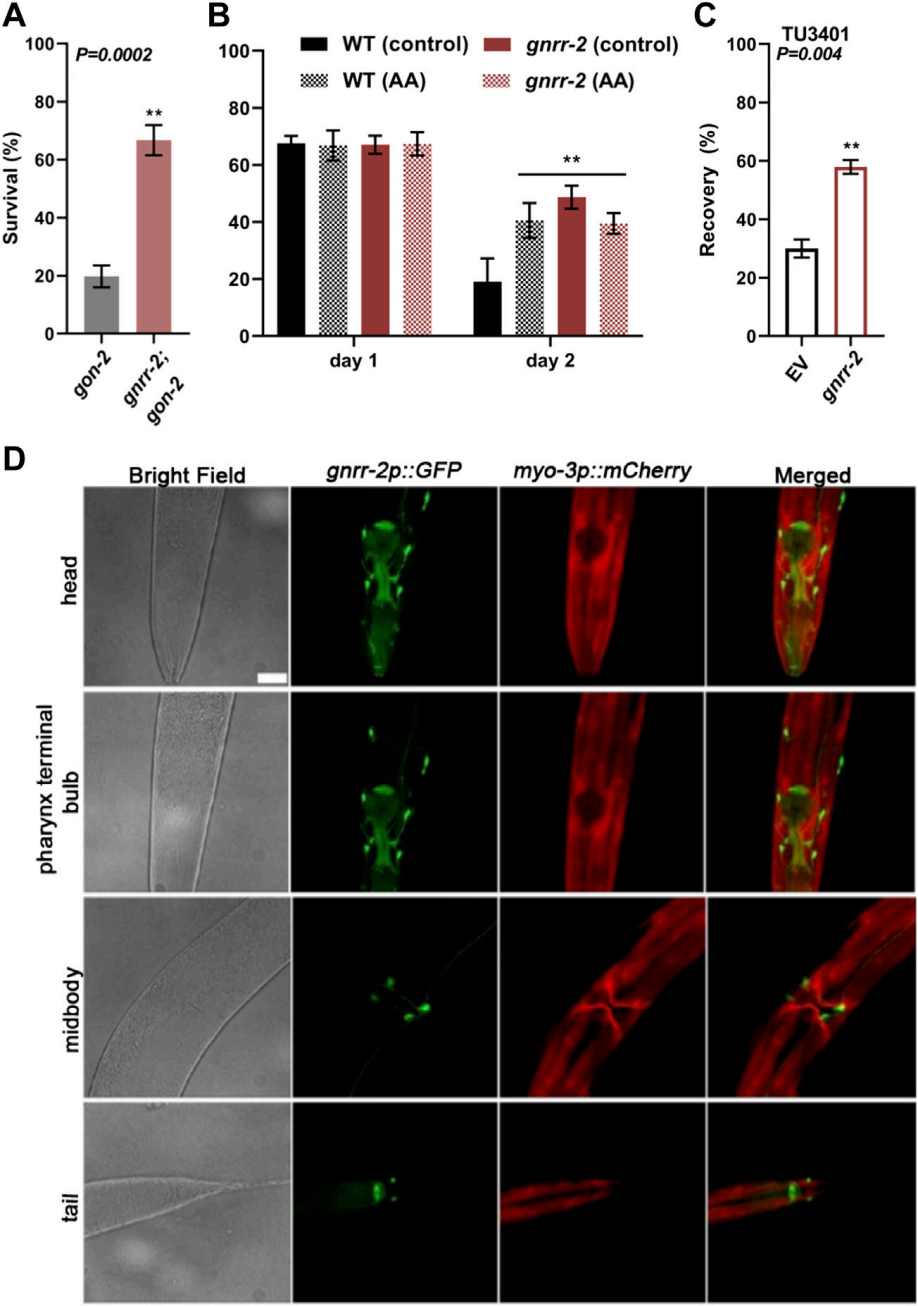
*gnrr-2* is expressed in the soma and function downstream of the gonad. **(A)** HS Survival rates of age-synchronized *gon-2* (*q388*) or *gnrr-2*;*gon-2* mutant animals. Day two adults were subjected to HS (6 h at 37°C), and survival was assayed (*N* ≥ 7). Data are means ±1 standard error of the mean (1 SE). Data were analyzed using the Wilcoxon Mann-Whitney rank sum test (*p* = 0.0002). **(B)** HS Survival rates of age-synchronized WT or *gnrr-2* mutant animals grown on control or arachidonic acid (AA) supplemented plates. Animals were subjected to HS (6 h at 37°C) on day one or two of adulthood, and survival was assayed (*N* ≥ 3). Data are means ±1 standard error of the mean (1 SE). Data were analyzed using one-way ANOVA followed by a Tukey’s *post-hoc* test. (**) denotes *p* ≤ 0.006 compared with same-age WT control animals. **(C)** HS recovery rates of age-synchronized neuronal RNAi hypersensitive animals fed on empty vector (EV) or *gnrr-2* RNAi-expressing bacteria. Day three adults were subjected to HS (4 h at 37°C), and recovery was assayed (*N* ≥ 5). Data are means ±1 standard error of the mean (1 SE). Data were analyzed using the Wilcoxon Mann-Whitney rank sum test (*p* = 0.004). **(D)** *gnrr-2* expression. Representative confocal Z-stack images of young adults expressing *gnrr-2p*::*GFP* and marker, *myo-3p*::*mCherry*. Ventral views of head, pharynx terminal bulb, midbody, and tail. The scale bar is 25 µm.

To ask whether *gnrr-2* mediates proteostasis collapse within the reproductive system or in the soma, we next asked whether mutant *gnrr-2* rescue of HS survival rate required the reproductive system. For that, we crossed *gon-2*(*q388ts*) mutant animals lacking the entire reproductive system with *gnrr-2* mutant animals and monitored thermotolerance. As shown previously, HS survival rates of *gon-2* mutant animals declined sharply on day two of adulthood (6 h at 37°C, 20% ± 4%), similar to WT (Shemesh et al., 2013). In contrast, the HS survival rate of *gnrr-2*;*gon-2* mutant animals was higher than *gon-2* (67% ± 5%, Wilcoxon Mann-Whitney rank sum test, *p* = 0.002; Figure 4A), similar to *gnrr-2* (Figure 1B). These data demonstrate that *gnrr-2* impact is downstream of the reproductive system. To further examine whether *gnrr-2* acts in the soma, we monitored the impact of arachidonic acid (AA) supplementation, which remodels somatic proteostasis (Shemesh et al., 2017b), on the thermotolerance of *gnrr-2* mutants. HS survival rate of *gnrr-2* mutant animals treated by AA was not further improved compared to control-treated animals and was similar to AA treated WT animals (day two adults, 6 h at 37°C, 39% ± 4%, 49% ± 4%, and 40% ± 6% respectively, compared to 19% ± 8% for control-treated WT, ANOVA followed by a Tukey’s post-hoc test; Figure 4B). Thus, mutant *gnrr-2* behaves similarly to a somatic modulator of the gonadal longevity pathway and does not require the gonad. Notably, *gnrr-2* differs from the embryo-to-mother signaling that requires the gonad and fertilized eggs to remodel somatic proteostasis (Sala et al., 2020). Taken together, our data suggest that *gnrr-2* functions in the soma downstream of the gonadal longevity signals.

We next ask whether GNRR-2 is expressed and functions in the soma (Figures 4C,D). We cloned GFP under the regulation of *gnrr-2* promoter (*gnrr-2p*::*GFP*) and examined the expression pattern of GFP. GFP was detected in the pharynx and specific neurons in the head, vulva, and tail regions (Figure 4D). Apart from the valval expression, GFP was observed throughout larval development (Supplementary Figure S4). Neuronal expression data from individual neurons supports this expression pattern (Taylor et al., 2021). For example, GFP expression was detected in the HSN vulval neurons showing *gnrr-2* expression. Thus, *gnrr-2* is expressed in the soma, mainly in neurons.

To determine whether proteostasis remodeling is associated with *gnrr-2* expression in neurons, we used a neuronal RNAi hypersensitive strain (TU3401) with a neuronal-specific expression of *sid-1* (Calixto et al., 2010). A transmembrane protein that enables passive uptake of dsRNA, and thus required for systemic RNAi. When HS recovery rates (4 h at 37°C, day three adults) were monitored, we observed an increased recovery for TU3401 animals treated with *gnrr-2*(*RNAi*) compared to EV control (58% ± 2% and 30 ± 3%, respectively, Wilcoxon Mann-Whitney rank-sum test, *p* = 0.004; Figure 4C). Thus, *gnrr-2* neuronal-expression can mediate proteostasis collapse.

### *gnrr-2* modulates reproduction

**FIGURE 5:**
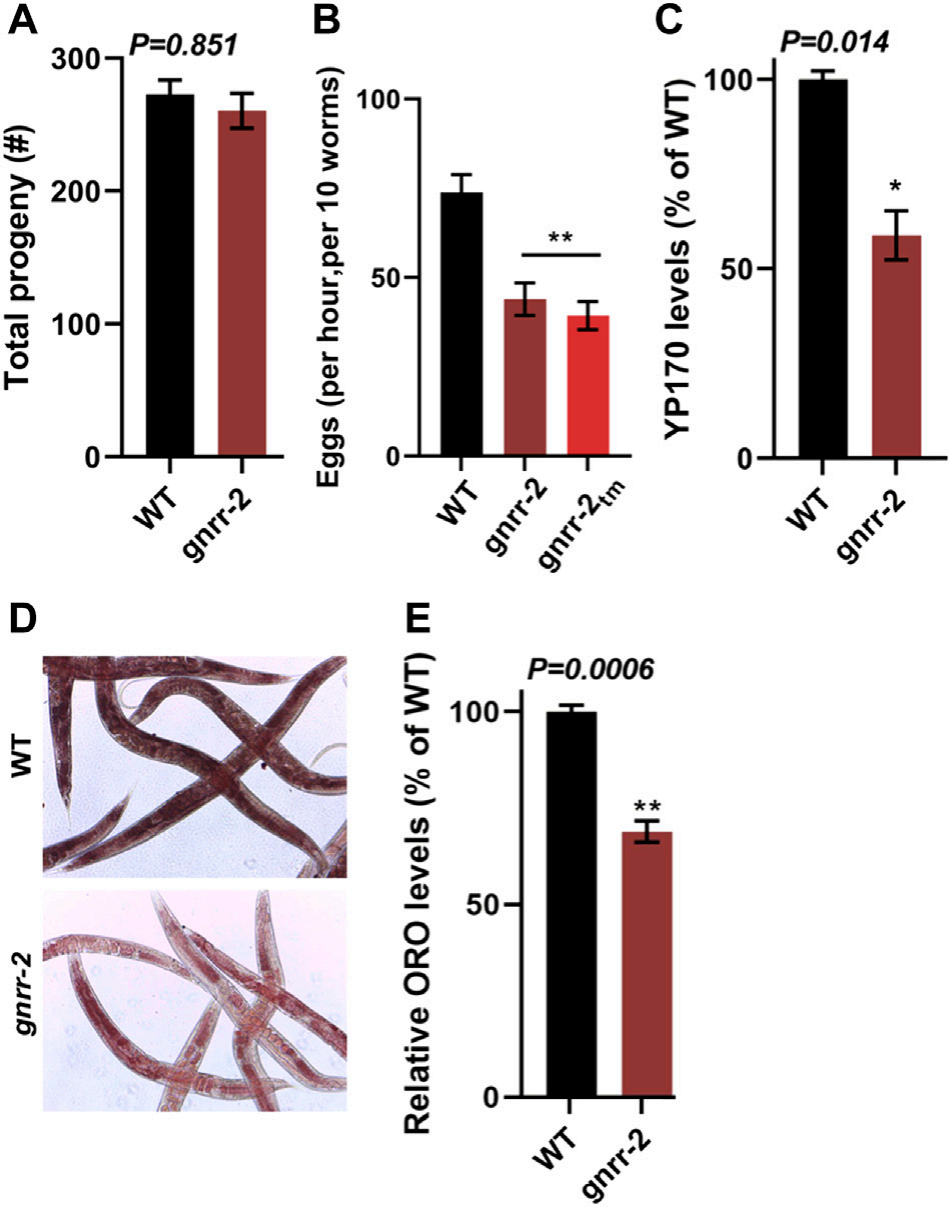
*gnrr-2* modulates reproduction. **(A)** Brood size of WT and *gnrr-2* animals. Progeny numbers were scored for age-synchronized fertile animals (*n* ≥ 18). Data are means ±1 standard error of the mean (1 SE). Data were analyzed using the Wilcoxon Mann-Whitney rank sum test (*p* = 0.851). **(B)** Egg-laying rates of WT, *gnrr-2*, and *gnrr-2*_*tm*_ animals. The number of eggs laid by ten age-synchronized day two adult animals per hour (*N* ≥ 11). Data are means ±1 standard error of the mean (1 SE). Data were analyzed using one-way ANOVA followed by a Tukey’s *post-hoc* test. (**) denotes *p* ≤ 0.001 compared with WT animals. **(C)** Vitellogenin levels in age-synchronized WT and *gnrr-2* animals. Extracts of age-synchronized day two adult animals were separated on an SDS-PAGE gel and yolk protein (YP170) levels were quantified (*N* = 4). Data are means ±1 standard error of the mean (1 SE). Data were analyzed using the Wilcoxon Mann-Whitney rank sum test (*p* = 0.014). **(D,E)** Representative images and quantification of total fat stores in WT and *gnrr-2* mutant animals. Total fat stores in age-synchronized day two adult animals were imaged using ORO staining **(D)**, and ORO levels of WT (*n* = 60) or *gnrr-2* (*n* = 71) mutant animals were quantified from the images *N* = 3 **(E)**. Data are means ±1 standard error of the mean (1 SE). Data were analyzed using the Wilcoxon Mann-Whitney rank sum test (*p* = 0.006).

GnRH-like signaling in invertebrates regulates various aspects of reproduction and associated reproductive treats (Sakai et al., 2020). Likewise, somatic activation of the gonadal longevity pathway is coupled with reproduction (Shemesh et al., 2017b). Thus, we next focused on the impact of *gnrr-2* on reproduction. The brood size of *gnrr-2* mutant animals was similar to WT (260 ± 13 and 272 ± 10, respectively, Wilcoxon Mann-Whitney rank sum test; Figure 5A). Likewise, embryo hatching and developmental timing were similar between WT and *gnrr-2* mutant strains (ANOVA followed by a Tukey’s post-hoc test; Supplementary Figures S5A–B). However, *gnrr-2* and *gnrr-2*_*tm*_ mutant animals showed a ∼1.7-fold reduction in the egg-laying rate (46 ± 4, 39 ± 4, and 71 ± 5 eggs per ten worms per hour, respectively, ANOVA followed by a Tukey’s post-hoc test, *p* ≤ 0.001; Figure 5B). In agreement with *gnrr-2* expression in HSN neurons that stimulate egg laying in hermaphrodites (Figure 4D). Moreover, RNAi hypersensitive (TU3401) animals treated with *gnrr-2*(*RNAi*) showed a mild (1.3-fold) reduction in the egg-laying rate compared to EV control, though TU3401 egg laying rate was also reduced (Wilcoxon Mann-Whitney rank-sum test, *p* = 0.046; Supplementary Figure S6A). A reduction in the egg-laying rate was also observed between the *gnrr-2*;*daf-16*, and *daf-16* mutant animals (30 ± 3 and 67 ± 9 eggs per ten worms per hour, respectively, ANOVA followed by a Tukey’s post-hoc test. *p* ≤ 0.001; Supplementary Figure S6B). In contrast, the egg-laying rates of *gnrr-2*;*pqm-1*, and *pqm-1* mutant animals (48 ± 6 and 45 ± 5 eggs per ten worms per hour, respectively) were similar to *gnrr-2* mutant animals (ANOVA followed by a Tukey’s *post-hoc* test; Supplementary Figure S6B). This observation suggests that *gnrr-*2-dependent modulation of PQM-1 function (Figures 3G–J) could impact the animals’ egg-laying rate.

GnRH-like signaling in invertebrates modulates vitellogenin (*vit*) gene expression (Gospocic et al., 2017; Nagel et al., 2020). *C. elegans* has six *vit* genes (*vit-1* to *vit-6*), mainly synthesized in the intestine and transported into the germline*.* Their expression is regulated in the intestine and is modulated by cell-nonautonomous signals from other tissues and in response to environmental cues (Ezcurra et al., 2018; Perez and Lehner, 2019; Sornda et al., 2019; Plagens et al., 2021). We, therefore, next examine the levels of VIT proteins in *gnrr-2* mutant animals. The levels of YP170 (the product of *vit-1* to *vit-5*) in *gnrr-2* mutant animals were reduced compared to WT (65% ± 8%, Wilcoxon Mann-Whitney rank sum test, *p* = 0.014; Figure 5C; Supplementary Figure S6C). Surprisingly, *vit-2* and *vit-3/4/5* mRNA (but not *vit-6*) levels were increased by ∼2-fold in *gnrr-2* mutant animals but not in *gnrr-2*;*daf-16* or *gnrr-2*;*pqm-1* mutant animals (ANOVA followed by a Tukey’s post-hoc test; Supplementary Figures S6D–F). These changes in expression suggest that disrupting *gnrr-2* dysregulates VIT production with a possible contribution from *daf-16* and *pqm-1*. We next compared fat stores in WT and *gnrr-2* mutant animals to complement this observation. Oil-Red-O (ORO) fatty acids staining in *gnrr-2* mutant animals was also reduced compared to WT (69% ± 3%, Wilcoxon Mann-Whitney rank sum test, *p* = 0.006; Figures 5D,E), further supporting *gnrr-2* impact on VIT production and fat accumulation. Of note, we observe a significant increase in Oil-Red-O staining in *gnrr-2*;*pqm-1* mutant animals (119% ± 3%, ANOVA followed by a Tukey’s post-hoc test. *p* ≤ 0.001; Supplementary Figure S6G). This increase further links PQM-1 to GNRR-2-dependent *vit* gene regulation.

## Discussion

### GNRR-2 is involved in the decision to commit to reproduction

**FIGURE 6:**
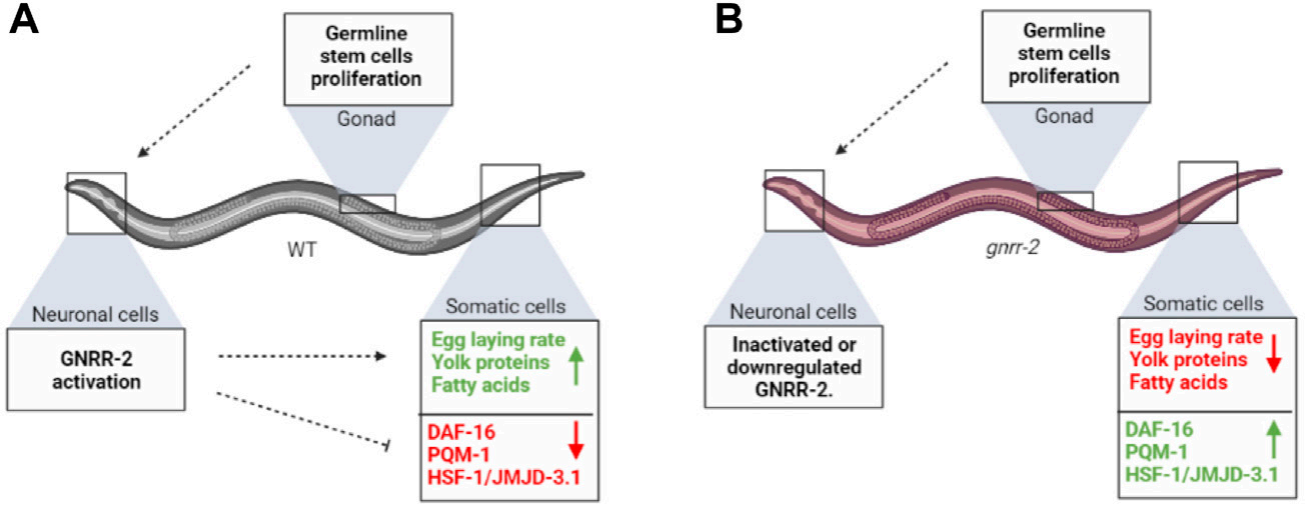
*gnrr-2* mediates signals from the reproductive system to the soma. **(A)** At the onset of WT oogenesis, gonadal signaling reports on GSCs’ competence to neuronal *gnrr-2*, who coordinately activates or represses various transcriptional programs in the soma, inversely modulating somatic maintenance and reproductive robustness. **(B)** Disrupting GNRR-2 function alters these signals, resulting in inhibition of proteostasis collapse and inhibition of somatic resources reallocation to the reproductive system. The figure was created using BioRender.com.

In *C. elegans*, a decision point at the onset of oogenesis regulates somatic proteostasis robustness in different somatic tissues (Shemesh et al., 2013; Labbadia and Morimoto, 2015). It also directs fat reserve usage to support reproduction by mobilizing fat stores from the intestine to the germline (Wang et al., 2008; Lapierre et al., 2011; Shemesh et al., 2017b; Dowen, 2019; Heimbucher et al., 2020). Here we asked whether GnRH-like signaling, which regulates reproduction and reproductive behaviors in various invertebrates (Zandawala et al., 2018; Sakai et al., 2020), promotes proteostasis remodeling. We identified a GnRH-like GPCR, *gnrr-2*, as a modifier of proteostasis in adulthood (Figures 1–3; Supplementary Figures S2–S3). *gnrr-2* also modulated egg-laying rates and vitellogenesis (Figure 5; Supplementary Figure S6). We determine that *gnrr-2* acts in the soma, specifically neurons (Figures 3, 4). We propose that neuronal *gnrr-2* responds to gonadal signaling and coordinates the somatic response to these signals, inversely modulating proteostasis and reproductive robustness, thus committing the organism to reproduction (Figure 6A). Other activating or repressing signals are likely to contribute to GNRR-2 function, as thermotolerance decline in gonad-less animals depends on GNRR-2 activity (Figure 4A). When GNRR-2 is inactivated or downregulated, gonadal (or other) signals are transmitted. However, they are no longer mediated to the soma, robust proteostasis is maintained, and resource transfer to progeny production is limited due to the modulation of different somatic transcriptional programs (Figure 6B). Our findings, therefore, support the emerging role of the nervous system, specifically neuropeptide signaling, in coordinating proteostasis across somatic tissues (Prahlad et al., 2008; Maman et al., 2013; Frakes et al., 2020; Hoppe and Cohen, 2020; Ozbey et al., 2020; Prahlad, 2020; Boocholez et al., 2022).

### GNRR-2 is required for remodeling proteostasis and reproduction at the onset of oogenesis

The decision to commit the organism to reproduction depends on nutrient availability, germline and embryo reproductive potency, and favorable environmental conditions (Maklakov and Immler, 2016; Aprison et al., 2022; Sala and Morimoto, 2022). This decision thus requires integrating internal and external signals to weigh the chances to reproduce successfully before reallocating metabolic resources (Antebi, 2013; Baugh and Hu, 2020; Gaddy et al., 2021; Aprison et al., 2022). Several at least partially independent pathways mediate proteostasis remodeling specifically in adulthood; gonadal longevity signaling reports on germline reproductive potency (Berman and Kenyon, 2006; Ermolaeva et al., 2013; Shemesh et al., 2017a; Yunger et al., 2017), an embryo-to-mother pathway reports on embryo integrity (Sala et al., 2020), and dietary signaling reports on nutrient availability (Tepper et al., 2013; Thondamal et al., 2014; Steinbaugh et al., 2015; Nakamura et al., 2016; Matai et al., 2019; Shpigel et al., 2019). The inhibition of proliferation or damage to germ cells results in DAF-16 nuclear localization and activation (Berman and Kenyon, 2006; Shemesh et al., 2017a; Yunger et al., 2017). It upregulates the expression of H3K27 demethylase *jmjd-3.1*, required for chromatin accessibility and HSF-1 transcriptional activation (Labbadia and Morimoto, 2015; Shemesh et al., 2017a). Likewise, disrupting embryo integrity activates DAF-16, albite only in vulval muscle (Sala et al., 2020). In contrast, dietary restriction requires *pqm-1* and modulates HSF-1 activation even late in life, independent from *jmjd-3.1* (Tepper et al., 2013; Shpigel et al., 2019). PQM-1 also mediates transcellular chaperone signaling that regulates inter-tissue proteostasis (O’Brien et al., 2018). Like gonadal signaling, mutations in *gnrr-2* resulted in upregulation of *jmjd-3.1* and required *jmjd-3.1* for HSR activation (Figure 3). Likewise, disrupting *gnrr-2* led to DAF-16 relocation to the nucleus and activation after the onset of reproduction. However, it also resulted in partial nuclear localization and activation of PQM-1 (Figure 3; Supplementary Figure S3). This dual requirement is unexpected because DAF-16 and PQM-1 function and localization are antagonistic (Tepper et al., 2013). However, *daf-16* and *pqm-1* are also required for insulin/IGF-1-like signaling and *ceh-60*-associated longevity (Tepper et al., 2013; Dowen, 2019). Moreover, both can regulate vitellogenesis (DePina et al., 2011; Dowen, 2019; Perez and Lehner, 2019; Heimbucher et al., 2020). Interestingly, DAF-16 could also modulate *gnrr-2* expression (Tepper et al., 2013). GNRR-2 activation may thus fine-tune DAF-16 and PQM-1 function to adjust somatic proteostasis and reproduction to various signals rather than act as an on/off switch.

GNRR-2 is expressed and potentially functions in neurons to regulate proteostasis and reproduction. Disrupting *gnrr-2* expression or function in gonad-less animals or specifically in neurons still remodeled proteostasis in adulthood (Figure 4). Moreover, arachidonic acid, a somatic regulator of the gonadal longevity signaling (Shemesh et al., 2017b), did not further improve *gnrr-2*-dependent HS survival rates. Likewise, the effect of GNRR-2 on egg-laying rate was in agreement with its expression in HSN neurons that stimulate egg-laying in hermaphrodites [and modulated by neuronal-specific *gnrr-2*(*RNAi*)]. Moreover, GNRR-2 modulated vitellogenin production, mainly synthesized in the intestine, and reduced YP170 protein levels and fat stores in *gnrr-2* mutant animals could be linked to DAF-16 and PQM-1 activation in the intestine. Nevertheless, other signaling pathways could also be involved (Wang et al., 2008; Dowen, 2019; Perez and Lehner, 2019; Sornda et al., 2019; Heimbucher et al., 2020). Thus, while GNRR-2 likely functions in neurons, tissue-specific expression of *gnrr-2* in the *gnrr-2* mutant background is needed to determine where GNRR-2 acts and whether proteostasis and reproduction regulation differ.

### GNRR-2 has a GnRH-like role, mediating reproduction-fitness trade-offs

In invertebrates, members of the GnRH superfamily neuropeptide signaling show pleiotropic activities (Zandawala et al., 2018; Sakai et al., 2020). Recently non-reproductive functions were also linked to GnRH and the pituitary gonadotropin, follicle-stimulating hormone, FSH, in vertebrates, including modulating Alzheimer’s disease-associated amyloid-β and Tau deposition (Skrapits et al., 2021; Xiong et al., 2022). However, many members regulate reproductive functions and metabolism, modulating reproduction-fitness trade-offs (Zandawala et al., 2018; Sakai et al., 2020). Specifically, members of the GnRH superfamily regulate brood size, egg-laying rates, vitellogenesis, mating behaviors, and even social reproductive behaviors such as cast identity in insects (Lindemans et al., 2009; Lebreton et al., 2016; Gospocic et al., 2017; Andreatta et al., 2020; Sakai et al., 2020). In *C. elegans*, there are eight members of this family, four of which were deorphanized. However, DAF-38/GNRR-8 functions with DAF-37 to mediate dauer entry in response to ascaroside pheromones (Park et al., 2012), while GNRR-3 and GNRR-6 regulate sleep and wakefulness in response to RPamide neuropeptides NLP-2 and NLP-22 (Van der Auwera et al., 2020). RPamide peptides share sequence similarity with GnRH/AKH peptides (Van der Auwera et al., 2020). However, only GNRR-1 was shown to respond to GnRH/AKH neuropeptide ortholog, NLP-47 (Lindemans et al., 2009). GNRR-1 is expressed in the nucleus of maturing oocytes and sperm cells and delayed egg-laying, supporting a GnRH-like role in modulating reproduction (Vadakkadath Meethal et al., 2006). But *gnrr-1* did not affect HS survival rates at the transition to adulthood (Figure 1). Thus, *gnrr* genes in *C. elegans* diverged both in function and peptide specificity.

The ability of a *C. elegans* peptide library to activate the GNRR-2 receptor was examined using an *in vitro* calcium mobilization assay, but no putative ligands were identified (Van der Auwera et al., 2020). Thus, while we find that GNRR-2 function is linked to GnRH-associated reproductive functions, such as egg-laying rates and vitellogenesis, it remains to be determined whether it responds to a GnRH neuropeptide ortholog. Likewise, the role of GNRR-6 in HSR modulation needs to be further examined, specifically whether this function is associated with sleep and wakefulness regulation by NLP-2 and NLP-22 or by a different neuropeptide. In this regard, it is interesting to note that GNRR-6 also responded to the FRPamide neuropeptide, NLP-23-2, *in vitro,* but *nlp-23* did not impact behavioral quiescence (Van der Auwera et al., 2020). NLP-23 could thus be a modulator of the HSR. Other neuropeptides were shown to modulate proteostasis (Frakes et al., 2020; Hoppe and Cohen, 2020; Ozbey et al., 2020; Prahlad, 2020; Boocholez et al., 2022) and might be linked to *gnrr-2*-depednent proteostasis remolding. Considering the conservation of proteostasis collapse (Sabath et al., 2020), understanding how non-autonomous signaling pathways integrate to modulate somatic proteostasis in *C. elegans* could offer novel approaches for treating age-dependent protein folding diseases. The findings that GnRH and FSH have non-reproductive functions and could modulate Alzheimer’s disease (Skrapits et al., 2021; Xiong et al., 2022), further stresses the importance of non-autonomous signaling pathways in regulating proteostasis.

## Data Availability

The raw data supporting the conclusion of this article will be made available by the authors, without undue reservation.
