# Supplementary material for: Gonadotropin-releasing hormone-like receptor 2 inversely regulates somatic proteostasis and reproduction in Caenorhabditis elegans
Source: Front Cell Dev Biol. 2022 Aug 29;10:951199. doi: 10.3389/fcell.2022.951199 (PMC9465036; doi:10.3389/fcell.2022.951199)
Supplement: Supplementary file 1 [file DataSheet1.pdf]

Supplementary Material

Gonadotropin-releasing hormone-like receptor 2 inversely regulates somatic proteostasis and reproduction in *Caenorhabditis elegans*

Mor Kishner, Libat Habaz, Lana Meshnik, Tomer Dvir Meidan, Alexandra Polonsky, Anat Ben-Zvi

1 Supplementary Figures

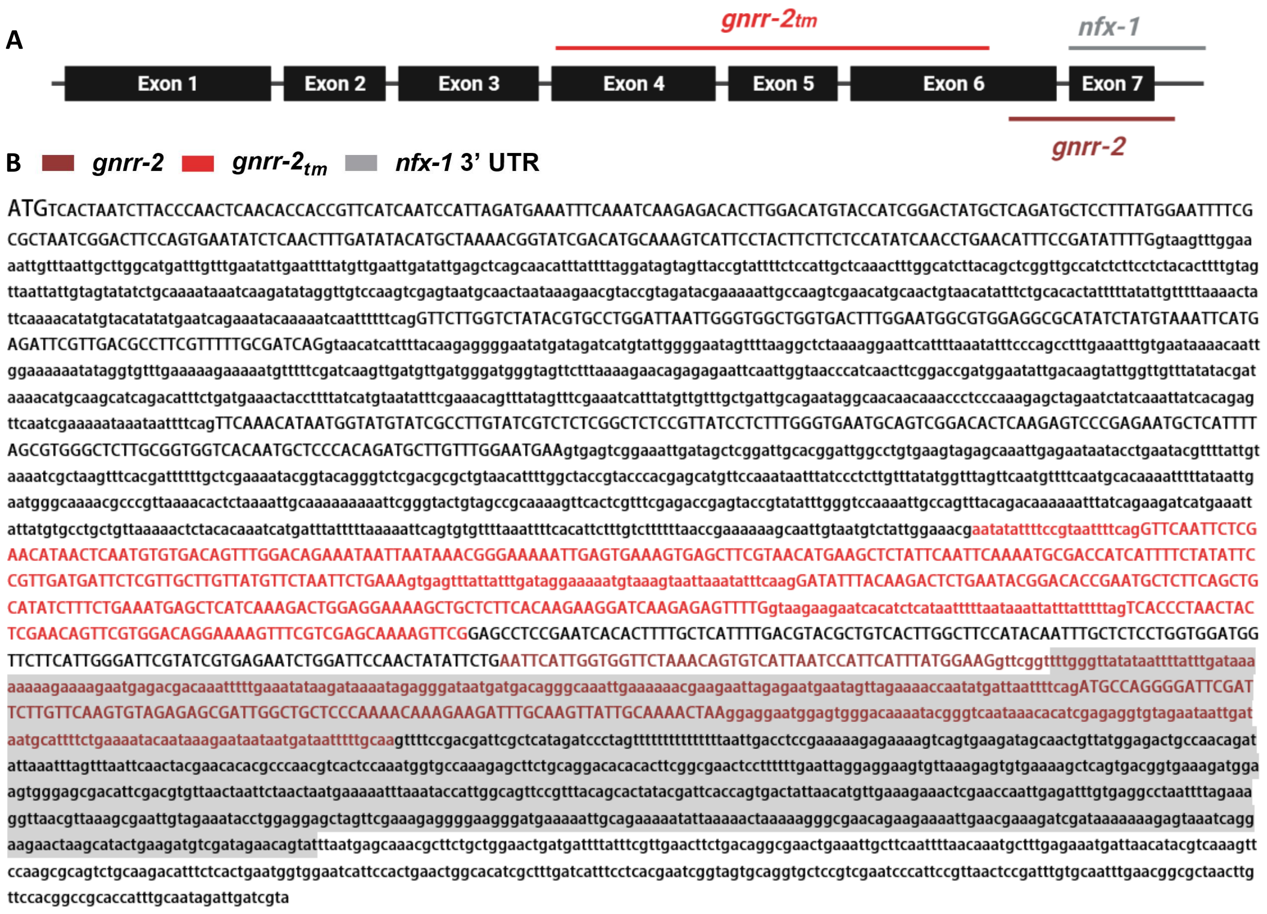

Figure S1. ***gnrr-2*** **deletion mutants. (A)** Genomic organization of the *gnrr-2* gene. The brown and red lines indicate the location of *ok3618* and *tm4867* deletion alleles, associated with *gnrr-2* and *gnrr-2*$_{tm}$, respectively. The gray line indicates the overlap between *gnrr-2* and *nxf-1* 3' UTR encoded on the opposite strand. **(B)** The unspliced sequence of the *gnrr-2* gene (from wormbase.com). The location of *ok3618* and *tm4867* deletions are marked in brown and red, respectively. A gray background marks the overlap between *gnrr-2* and *nxf-1* 3' UTR sequences.

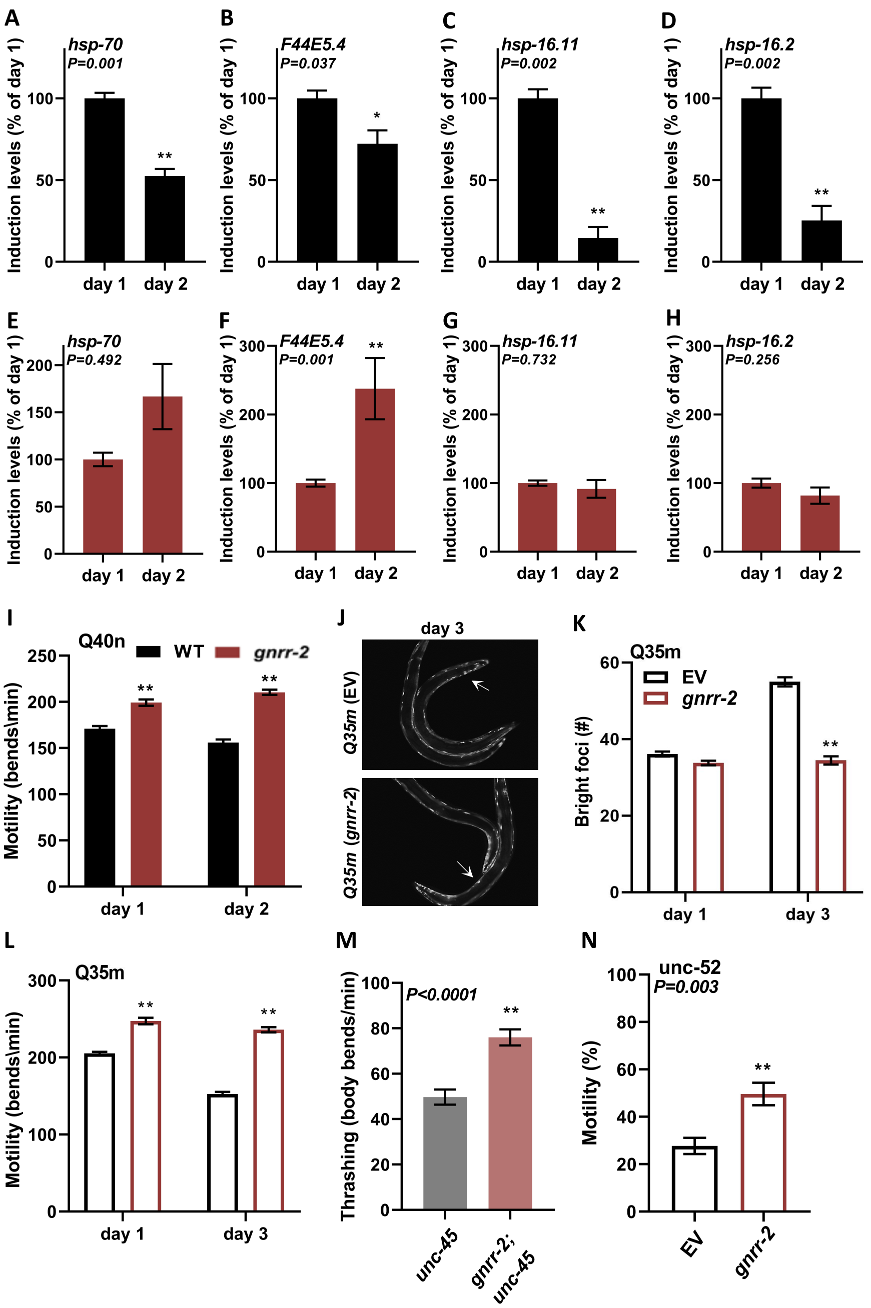

Figure S2. Disrupting *gnrr-2* expression rescues proteostasis in adulthood. (A-H) Expression levels of HS genes. mRNA levels of *hsp-70* (A, E)*, F44E5.4* (B, F)*, hsp-16.11* (C, G) and *hsp-16.2* (D, H) from age-synchronized day one or two adult WT (A-D) or *gnrr*-2 (E-H) animals subjected to HS (90 min at 37°C; N≥6). Data are means ± 1 standard error of the mean (1SE). Data were analyzed using the Wilcoxon Mann-Whitney rank sum test. **(I)** PolyQ-associated toxicity of age-synchronized WT or *gnrr-2(ok3618)* animals expressing *Q40n*. Thrashing rates were scored on day one or two of adulthood (n≥50). Data are means ± 1 standard error of the mean (1SE). Data were analyzed using one-way ANOVA followed by a Tukey's *post-hoc* test. (**) denotes *P*<0.001 compared with same-age WT animals expressing *Q40n*. **(J-L)** PolyQ-associated foci accumulation and toxicity of age-synchronized *Q35m* animals fed on empty vector (EV) control or *gnrr-2* RNAi-expressing bacteria. Age-synchronized *Q35m* expressing animals were imaged on day three of adulthood (J). Arrows indicate foci. The number of visible foci (n>50; K) or thrashing rate (n≥60; L) was scored on day one or three of adulthood. Data are means ± 1 standard error of the mean (1SE). Data were analyzed using one-way ANOVA followed by a Tukey's *post-hoc* test. (**) denotes *P*<0.001 compared with same-age *Q35m* expressing animals fed on EV. **(M)** Motility of age-synchronized *unc-45(e286ts)* or *gnrr-2;unc-45(ts)* animals. Thrashing rates were scored on day two of adulthood (n≥18). Data are means ± 1 standard error of the mean (1SE). Data were analyzed using the Wilcoxon Mann-Whitney rank sum test (*P*<0.001). **(N)** Stiff body paralysis of age-synchronized *unc-52(e669su250ts)* or *gnrr-2;unc-52(ts)* animals. The percent of paralyzed animals was scored on day four of adulthood (N≥12). Data are means ± 1 standard error of the mean (1SE). Data were analyzed using the Wilcoxon Mann-Whitney rank sum test (*P*=0.003).

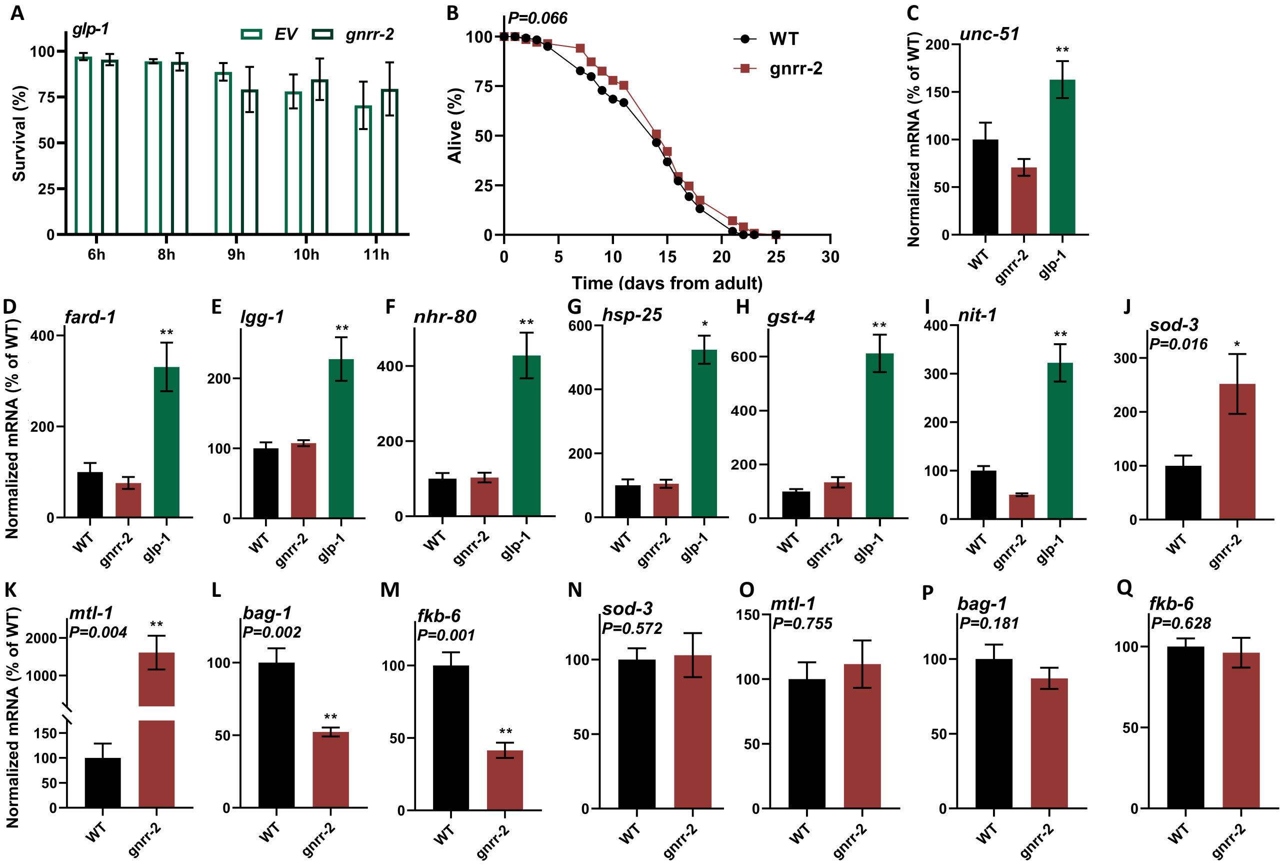

Figure. S3. ***gnrr-2*** **functions in the gonadal longevity pathway.** **(A)** Survival rates of age-synchronized *glp-1* animals fed on empty vector (EV) control or *gnrr-2* RNAi-expressing bacteria. Animals were subjected to HS (6-11 h, as indicated, at 37°C) on day two of adulthood and survival was assayed (N=5). Data are means ± 1 standard error of the mean (1SE). Data were analyzed using one-way ANOVA followed by a Tukey's *post-hoc* test. **(B)** Lifespan of WT or *gnrr-2* animal. Age-synchronized WT 130 (15 censored) or *gnrr-2* 145 (16 censored) were monitored every day, and the number of dead or censored animals was determined. Mean life spans (WT 16.5±0.5 and *gnrr-2* 17.6±0.4) were calculated using Kaplan-Meier survival curves. Data were analyzed using the log-rank Mantel-Cox test (*P*=0.066). **(C-I)** Expression levels of *glp-1*-associated transcription factors' targets in age-synchronized WT, *gnrr-2,* or *glp-1* animals. mRNA was extracted from day two adult animals, and mRNA levels of *unc-51* (C), *fard-1* (D), *lgg-1* (G), *nhr-80* (F), *hsp-25* (G), *gst-4* (H), and *nit-1* (I) were quantified (N≥5). Data are means ± 1 standard error of the mean (1SE). Data were analyzed using one-way ANOVA followed by a Tukey's *post-hoc* test. (*) denotes $P<0.05$ and (**) denotes $P<0.01$ compared with same-age WT animals. **(J-Q)** Expression levels of DAF-16 or PQM-1 associated targets in age-synchronized WT or *gnrr-2* animals. mRNA was extracted from day two adults subjected to HS (90 min at 37°C; J-M) or from day one adults (N-Q) and levels of *sod-3* (J, N), *mtl-1* (K, O), *bag-1* (L, P), and *fkb-6* (M, Q) were quantified (N≥4). Data are means ± 1 standard error of the mean (1SE). Data were analyzed using Wilcoxon Mann-Whitney rank sum test.

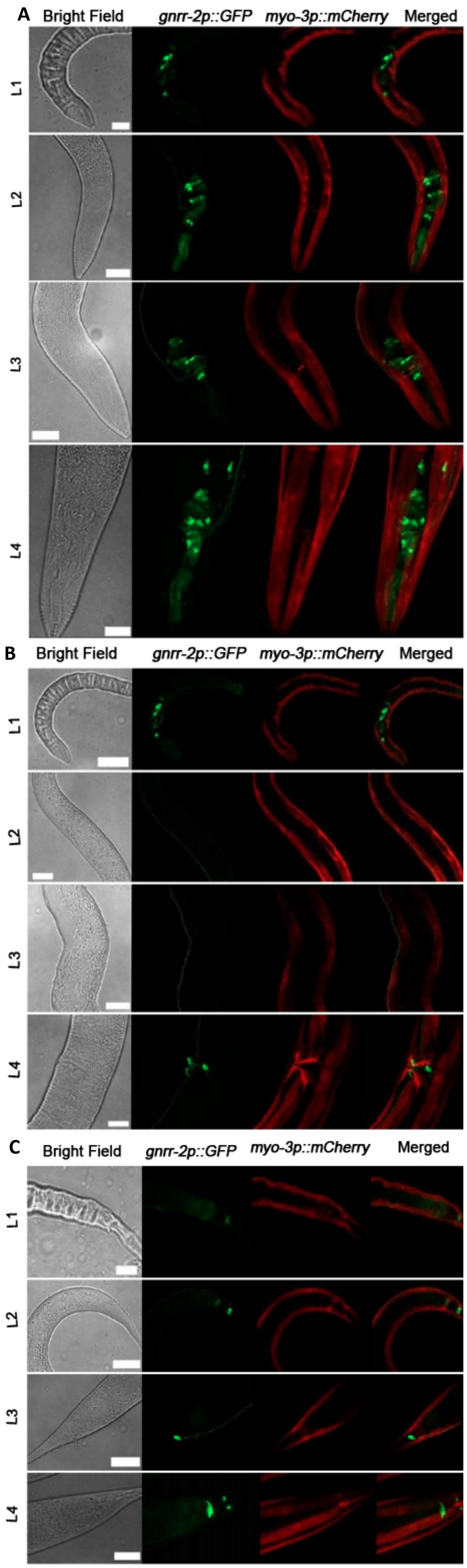

Figure. S4. *gnrr-2p::GFP* expression during development. (A-C) Representative confocal Z-stack images of the head (A), midbody (B), or tail (C) of larvae stages 1-4 (L1-4) expressing *gnrr-2p::GFP* and *myo-3p::mCherry* marker. Scale bars are 25 µm.

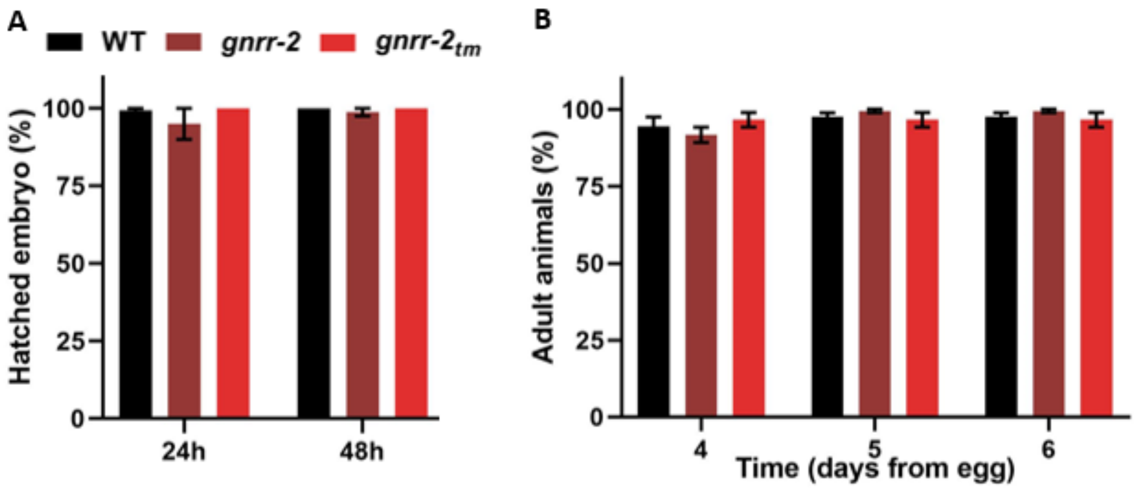

Figure. S5. *gnrr-2* does not modulate embryo hatching and developmental timing. (A-B) Embryo hatching and developmental timing of WT, *gnrr-2, or gnrr-2$_{tm}$* mutant animals. The percent of WT, *gnrr-2,* or *gnrr-2$_{tm}$* mutant embryos that hatched 24-48 h after being laid (A) and reached reproductive adulthood after 4-6 days (B) was scored (N>4). Data are means ± 1 standard error of the mean (1SE). Data were analyzed using one-way ANOVA followed by a Tukey's *post-hoc* test.

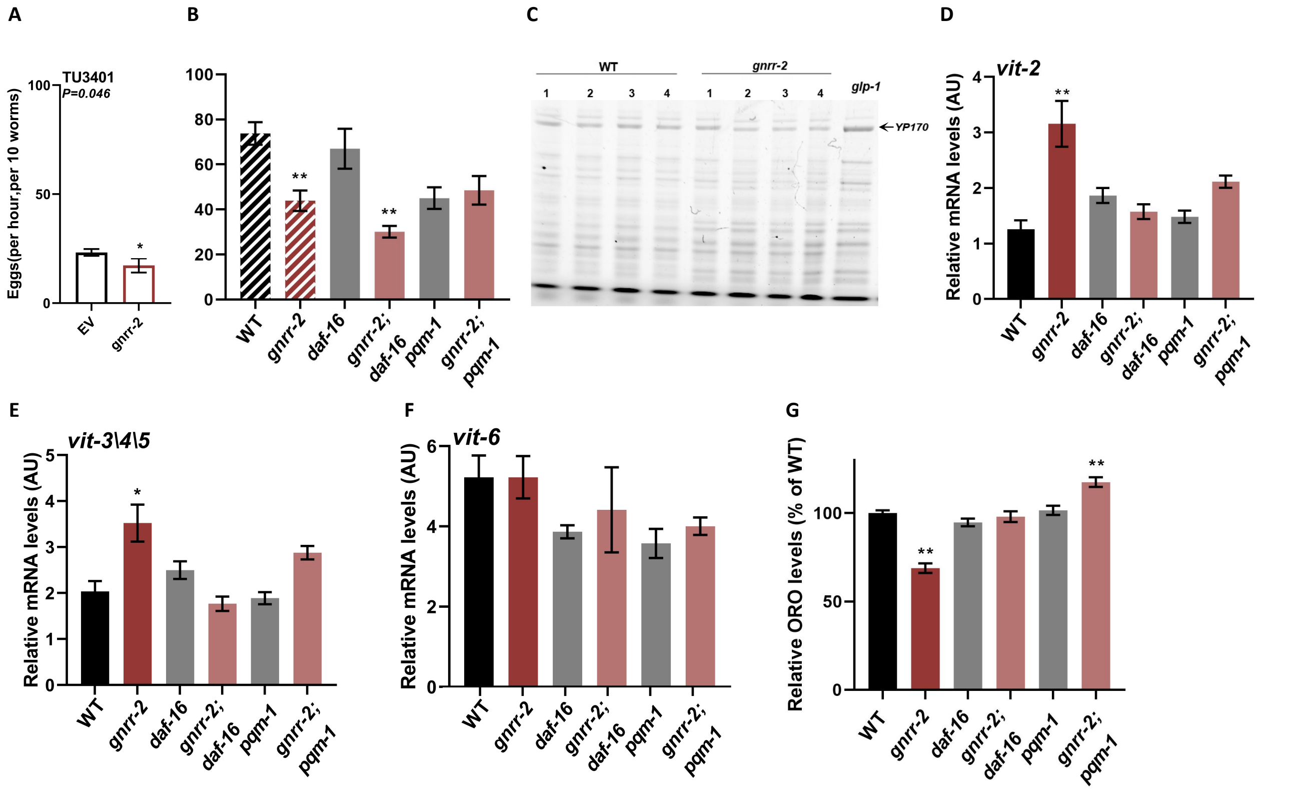

Figure. S6. The impact of *daf-16* and *pqm-1* on *gnrr-2*-dependent modulation of reproduction. **(A)** Egg-laying rates of age-synchronized neuronal RNAi hypersensitive animals fed on empty vector (EV) or *gnrr-2* RNAi-expressing bacteria. The number of eggs laid by ten age-synchronized day two adult animals per hour (N≥7). Data are means ± 1 standard error of the mean (1SE). Data were analyzed using one-tailed Wilcoxon Mann-Whitney rank sum test (*P*=0.046). **(B)** Egg-laying rates of WT and *gnrr-2* mutant animals, as indicated. The number of eggs laid by ten age-synchronized day two adult animals per hour (N≥11). Data are means ± 1 standard error of the mean (1SE). Data were analyzed using one-way ANOVA followed by a Tukey's *post-hoc* test. (**) denotes $P \leq 0.002$ comparing with the relevant *gnrr-2* background. **(C)** Image of SDS-PAGE gel used for quantification of vitellogenin. Extracts of age-synchronized day two adult WT, *gnrr-2* and *glp-1* animals were separated on SDS-PAGE gel. Yolk protein, YP170, is indicted with an arrow. **(D-F)** Expression levels of *vit* genes in age-synchronized animals, as indicated. mRNA was extracted from day two adult animals, and mRNA levels of *vit-2* (D), v*it-3,4,5* (E), and v*it-6* (F) were quantified (N≥5). Data are means ± 1 standard error of the mean (1SE). Data were analyzed using one-way ANOVA followed by a Tukey's *post-hoc* test. (*) denotes $P \leq 0.05$ (**) denotes $P \leq 0.001$ comparing with the relevant *gnrr-2* background. **(G)** Quantification of total fat stores in WT and *gnrr-2* mutant animals, as indicated. Total fat stores in age-synchronized day two adult animals were imaged using Oil-Red-O (ORO) staining and ORO levels of WT, *gnrr-2, daf-16, daf-16;gnrr-2, pqm-1* and *pqm-1;gnrr-2* mutant animals (n=60, 71, 43, 42, 44, and 39, respectively; N=3) were quantified from the images. Data are means ± 1 standard error of the mean (1SE). Data were analyzed using one-way ANOVA followed by a Tukey's *post-hoc* test. (**) denotes $P \leq 0.001$ compared with same-age WT animals.

2 Supplementary Tables

Strain	Abbreviation	Genotype
N2	Wild Type (WT)	---
RB509	*gnrr-1*	*gnrr-1(ok238)*
RB2596	*gnrr-2*	*gnrr-2(ok3618)*
tm4867	*gnrr-2$_{tm}$*	*gnrr-2(tm4867)*
tm4152	*gnrr-3*	*gnrr-3(tm4152)*
tm4218	*gnrr-4*	*gnrr-4(tm4218)*
tm4160	*gnrr-5*	*gnrr-5(tm4160)*
RB2439	*gnrr-6*	*gnrr-6(ok3362)*
tm4155	*gnrr-7*	*gnrr-7(tm4155)*
RB2090	*gnrr-8*	*gnrr-8(ok2765)*
ABZ204	*gnrr-2p::GFP*	*bguEx1[pgnrr-2::gfp]*
Cl2070	*GFP$_{HS}$*	*dvIs70[phsp-16.2::gfp; rol-6(su1006)]*
ABZ264	*gnrr-2;GFP$_{HS}$*	*gnrr-2(ok3618);dvIs70[phsp-16.2::gfp; rol-6(su1006)]*
ABZ307	*gnrr-2$_{tm}$;GFP$_{HS}$*	*gnrr-2(tm4867);dvIs70[phsp-16.2::gfp; rol-6(su1006)]*
AM140	*Q35m*	*rmIs132[punc-54::q35::yfp]*
ABZ265	*gnrr-2;Q35m*	*gnrr-2(ok3618);rmIs132[punc-54::q35::yfp]*
ABZ266	*gnrr-2$_{tm}$;Q35m*	*gnrr-2(tm4867);rmIs132[punc-54::q35::yfp]*
AM47	*Q40n*	*rmls167[pF25B3.3::q40::cfp]*
ABZ230	*gnrr-2;Q40n*	*gnrr-2(ok3618);rmls167[pF25B3.3::q40::cfp]*
CB286	*unc-45(ts)*	*unc-45(e286)*
ABZ260	*gnrr-2;unc-45(ts)*	*gnrr-2(ok3618);unc-45(e286)*
HE250	*unc-52(ts)*	*unc-52(e669su250)*
CF2253	*gon-2*	*gon-2(q388ts)*
ABZ209	*gnrr-2;gon-2*	*gnrr-2(ok3618);gon-2(q388ts)*
CF1903	*glp-1*	*glp-1(e2144ts)*
ABZ210	*gnrr-2;glp-1*	*gnrr-2(ok3618);glp-1(e2144ts)*
CF1038	*daf-16*	*daf-16(mu86)I*
ABZ248	*gnrr-2:daf-16*	*gnrr-2(ok3618);daf-16(mu86)*
RB711	*pqm-1*	*pqm-1(ok485)*
ABZ262	*gnrr-2:pqm-1*	*gnrr-2(ok3618);pqm-1(ok485)*
ZR2	*jmjd 3.1*	*jmjd-3.1(gk384)*
ABZ267	*gnrr-2:jmjd 3.1*	*gnrr-2(ok3618); jmjd-3.1(gk384)*
PS3551	*hsf-1*	*hsf-1(sy441)*
ABZ302	*gnrr-2:hsf-1*	*gnrr-2 (ok3618);hsf-1(sy441)*
TU3401	TU3401	*sid-1(pk3321);uIS69[Punc-119::sid-1;pCFJ90 (Pmyo-2::mCherry)]*

Table S1. List of strains used in this work. Strains were out-crossed at least three times to our laboratory N2 stock.

Target	F' sequence	R' sequence
gnrr-2	TGGGTCCAAAATTGCCAGT	ATCTATGAGCGAATCGTCGG
gnrr-2$_{tm}$	ATGGGCAAAACGCCCGTTAA	GAATCGAATCCCCTGGCATC
gnrr-3	TTCCGTAACTTAGGCTCCTC	CATACTCGACTATCCGTGAT
gnrr-4	GGAGGTTTGTATGACGTTAC	TCGGCAAAGAAGGGTACTTA
gnrr-5	CGTTCCTCCTTTGAAGTCGT	ACATGACACGCGAACCGCAG
gnrr-6	TCTTGACCTACTCCCTCGGT	ACCTCGACGGTTTCTTGCTA
gnrr-7	CCCCGGAGAAAAAACTAGGA	GGTATCTTTCGGTGTACAGT
gnrr-8	CAGAGCTCCTCCAACGTGAT	TTTCTGAATTTTTACCTGTTTTTGA
gnrr-2p	TCCAATTCCCATTACTCCCAAAGT	TTCTGGAATTAATACTTTGAAAATA
Pgnrr-2	TCACCATGGGAAGCTTCGTGGATC	GGGACAACTCCAGTGAAAAGTTCTT
pNU345	CTGGAGCCGGTGAGCGTGGGTCT	CCTGCAGGATATCTGGATCCACGAA
pNU345	ATGAGTAAAGGAGAAGAACTTTT	CTATCTCAGCGATCTGTCTATTTCG
pNU345	---	CAGTAGTGCAAATAAATTTAAGG
actin	ATCACCGCTCTTGCCCCATC	GGCCGGACTCGTCGTACTCTTG
18S	GCCAGCAGCCGCGGTAATTCCAG	TTGCGAATCTGAGGCACGTAACCT
hsp-70	CTACATGCAAAGCGATTGGA	TGTAAAAGCCACGTAGGAAGG
F44E5.4	GCAAAGCTATTGGTATCGAC	CACGTATGATGGAGTTGTCTTA
hsp16.11	CCACTATTTCCGTCCAGCTC	GCTTGAACTGCGAGACATTG
hsp16.2	ACTTTACCACTATTTCCGTCCAGC	CCTTGAACCGCTTCTTTCTTTG
daf-16	TTCCGTCTGCGTTTCTTTCT	AACCGGAAAGATGATGGAAC
daf-16	GTCCTCATTCACTCCCGATT	CTGTCGACCCGTTTGAAGAT
pqm-1	GGTTCTCCATTGTGTGCTCA	ACAACCCTCAATTTTGCAGG
pqm-1	ATTCCGTGCCGATAGTTCTT	AACCCCTACCTGATGCAAAA
jmjd-3.1	CAGTTTGTTCCCCAGCATTT	TTCGCTTTGGCGGTACTACT
Jmjd-3.1	ACGCCTGATGTTCTCATTCGCA	TATGCTGCACAGGCACAGGTTTC
hsf-1	AACAAATCCTCGGCTCCATCAT	AATTGGGCGGGGTTTGCTG
sod-3	GCTGCAATCTACTGCTCGCACTG	GGCTGATTACAGGTTCCAAATCTGC
mtl-1	ATGGCTTGCAAGTGTGACT	CAGTCTCCCTTACATCCAGC
bag-1	AAGAGGCGGAAAGACATCTG	GATTCCGTTTACGAGCGTCT
fkb-6	AGATCGCGGTGATCAATTCT	AATCCGAGCGAATTGTGAAT
unc-51	AATGAGCCGTTGGATAATGC	GAGGAGGTTGAGGATGTGGA
fard-1	CATATACCTGCTGCCATCTCA	CAGTCCACGTGCATTGAAAC
lgg-1	CATTCCACAAACCATGACCA	TCTTTTCGACCTCTCCTCCA
nhr-80	TCCAGCTGCTCAACCTCG	AAGCCCAAAAATTCATTTATTC
hsp-25	GTCGTGTTGAGGAGGAGAT	AGAAGAAGTGTTCGAGTAGGC
gst-4	CCCATTTTACAAGTCGATGG	CTT CCT CTG CAG TTT TTC CA
nit-1	AATCCTCCGACTATCCCTTG	AGC GAA TCG TTT CTT TTG TG
vit-2	AAAGCTCGTTCTTGAGGAGA	CTCAGCCTTGTCTCCATTCT
vit-3,4,5	CATGTGCACCATCGAAGAACTC	CCAATGTGGTTTCAATGACAAGTTG
vit-6	TTCACCCAGAAGCCAGTTC	AGG ATG GGA GGC AGT AGA C

Table S2. List of primers used in this work
